# Feasibility of a dietary intervention to modify gut microbial metabolism in patients with hematopoietic stem cell transplantation

**DOI:** 10.1038/s41591-023-02587-y

**Published:** 2023-10-19

**Authors:** Mary M. Riwes, Jonathan L. Golob, John Magenau, Mengrou Shan, Gregory Dick, Thomas Braun, Thomas M. Schmidt, Attaphol Pawarode, Sarah Anand, Monalisa Ghosh, John Maciejewski, Darren King, Sung Choi, Gregory Yanik, Marcus Geer, Ethan Hillman, Costas A. Lyssiotis, Muneesh Tewari, Pavan Reddy

**Affiliations:** 1 Department of Internal Medicine, Division of Hematology and Oncology, University of Michigan, Rogel Cancer Center, Ann Arbor, MI USA; 2 Department of Internal Medicine, Division of Infectious Disease, University of Michigan, Ann Arbor, MI USA; 3 Department of Molecular & Integrative Physiology, University of Michigan, Ann Arbor, MI USA; 4 Department of Earth & Environmental Sciences, University of Michigan, Ann Arbor, MI USA; 5 Department of Biostatistics, University of Michigan, Ann Arbor, MI USA; 6 Dan L Duncan Comprehensive Cancer Center, Baylor College of Medicine, Houston, TX USA

**Keywords:** Translational research, Microbial communities

## Abstract

Evaluation of the impact of dietary intervention on gastrointestinal microbiota and metabolites after allogeneic hematopoietic stem cell transplantation (HCT) is lacking. We conducted a feasibility study as the first of a two-phase trial. Ten adults received resistant potato starch (RPS) daily from day −7 to day 100. The primary objective was to test the feasibility of RPS and its effect on intestinal microbiome and metabolites, including the short-chain fatty acid butyrate. Feasibility met the preset goal of 60% or more, adhering to 70% or more doses; fecal butyrate levels were significantly higher when participants were on RPS than when they were not (*P* < 0.0001). An exploratory objective was to evaluate plasma metabolites. We observed longitudinal changes in plasma metabolites compared to baseline, which were independent of RPS (*P* < 0.0001). However, in recipients of RPS, the dominant plasma metabolites were more stable compared to historical controls with significant difference at engraftment (*P* < 0.05). These results indicate that RPS in recipients of allogeneic HCT is feasible; in this study, it was associated with significant alterations in intestinal and plasma metabolites. A phase 2 trial examining the effect of RPS on graft-versus-host disease in recipients of allogeneic HCT is underway. ClinicalTrials.gov registration: NCT02763033.

## Abstract (web summary)

In a pilot feasibility study involving recipients of allogeneic hematopoietic stem cell transplants, adherence to a dietary intervention and its effects on the gut microbiota were assessed in the run-in to a study testing the effects of modulation of the microbiome on transplant outcomes.

## Main

Interactions between the intestinal microbiome and the host’s immune and nonimmune cells are crucial influences on health and disease^1,2^. These interactions depend critically on nutrient processing and production of metabolites by the intestinal microbiota; however, evidence that dietary interventions that modulate the intestinal microbiome can alleviate the course of clinical diseases is limited^3^. Emerging data suggest that alterations in the intestinal microbiota and its metabolites have an important role in modulating the severity of acute gastrointestinal (GI) graft-versus-host disease (GVHD) after allogeneic hematopoietic stem cell transplantation (HCT)^4–7^. Allogeneic HCT is a potentially curative treatment modality for patients with malignant and benign hematological and inherited diseases^8,9^. GVHD is the principal cause of non-relapse mortality and the major cause of morbidity after allogeneic HCT^10^. Acute GVHD results from an allogeneic immune response driven by donor T cells and most commonly affects the host skin, liver and GI tract^11,12^. Recent reports implicated changes in the structure of the intestinal microbiome as a major contributor to GVHD severity and outcomes^13–16^. However, data on rationally and prospectively modifying the intestinal microbiome to decrease human GVHD is limited except for use of fecal microbiota transplantation (FMT)^17^. However, FMT faces challenges including safety concerns in immunocompromised patients, limitations in scalability for widespread implementation and as yet unclear efficacy^18^. A safer and more practical approach to modify the intestinal microbiome is via dietary prebiotics^19^. Prebiotics are forms of starch that resist degradation by host enzymes but are metabolized by specific gut bacteria and alter microbiome-derived metabolite composition^20–22^. However, it is unknown if prebiotic intervention in patients with allogeneic HCT can rationally modify the intestinal microbiome for therapeutic benefit^23^.

The severity of acute GI GVHD is associated with a shift toward *Enterococcus* or oral microbiome organisms in place of gram-positive obligate anaerobic bacteria typically found in the healthy gut and a reduction in specific microbial metabolites, especially short-chain fatty acids (SCFAs) such as butyrate, made by these same gram-positive anerobes^24–28^. SCFAs (for example, butyrate, acetate and propionate) are the most studied microbial metabolites^29^. They are produced from the fermentation of indigestible carbohydrates by intestinal anaerobic commensal bacteria such as *Clostridia* species and serve as an energy source for the intestinal microbiota and the host’s intestinal epithelial cells (IECs)^29^, and are essential for the maintenance of the intestinal mucosal barrier^30,31^. Experimental data demonstrated that butyrate was significantly decreased in the IECs of mice experiencing GVHD, while restoring butyrate levels by increasing intestinal butyrate-producing bacteria reduced experimental acute GI GVHD severity and mortality^32^. Administration of defined quantities of resistant potato starch (RPS), as a prebiotic, to normal healthy human volunteers promoted an increase in butyrogenic bacteria and intestinal levels of butyrate to a greater extent than other commercially available resistant starch (RS) preparations tested^33,34^. Based on these data, we hypothesized that administration of a defined quantity of RPS in patients with allogeneic HCT would be feasible and promote butyrogenic microbiota, increasing levels of salutary metabolites such as butyrate, despite the use of several HCT-related medications, including antibiotics.

## Results

### Administration of RPS is feasible and tolerated by patients

**Fig. 1:**
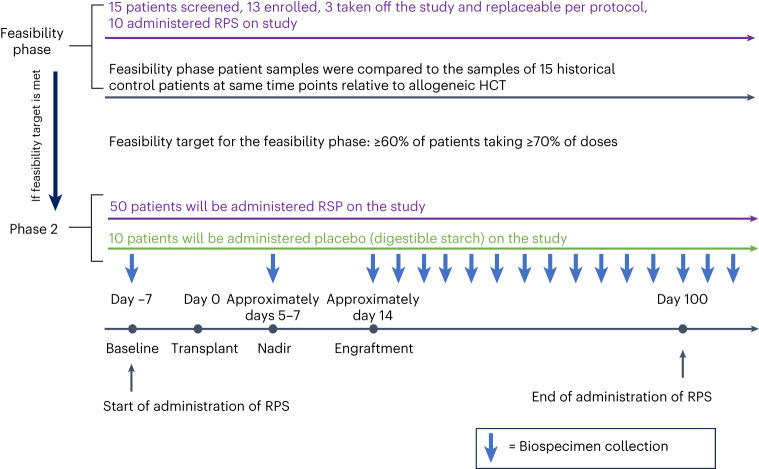
Schema for the clinical trial and longitudinal biospecimen collection. Study participants received 20 g RPS orally daily for the first 3 days followed by twice daily, from day −7 through to day 100 after allogeneic HCT. Stool and blood specimens were collected from the study participants at baseline before conditioning (day −7), nadir (approximately days 5–7), engraftment (approximately day 14) and day 100. Stool samples were collected weekly after allogeneic HCT when possible. Samples were compared to samples obtained from past historical controls not receiving the dietary intervention at the same time points relative to allogeneic HCT. After the feasibility phase, an additional 50 individuals will be enrolled on phase 2 of the trial to receive RPS on the same schedule noted above and ten individuals will be enrolled to receive isocaloric, nonresistant starch placebo, who will serve as contemporaneous controls (5:1 randomization). Stool and blood specimens will be collected on the same schedule as the feasibility phase.

We performed a single-center prospective, single-arm, longitudinal study between 26 April 2017 and 30 September 2018 as the first part of a phase 2 clinical trial (NCT02763033). We recruited adults who were undergoing human leukocyte antigen (HLA) matched related donor (MRD) myeloablative allogeneic HCT. Study participants received 20 g RPS orally, produced by Bob’s Red Mill and packaged by research pharmacy, daily for the first 3 days followed by twice daily, from day −7 through day 100 after allogeneic HCT (Fig. 1). We designed RPS administration to start daily before increasing to twice daily for patients to get used to it and be more likely to tolerate it given that these patients experience a lot of GI side effects from their conditioning regimens.

Patients with allogeneic HCT often suffer from treatment-related GI distress and GVHD, thus making dietary interventions difficult^35,36^. Therefore, we assessed whether stringent administration of RPS as a dietary intervention was clinically feasible and tolerable by the patients. Ten individuals were enrolled and administered RPS on the study (Table 1). We preset a target for feasibility for 60% or more patients to adhere to 70% or more of scheduled doses. The median age was 57 years (range: 52–62 years). All participants received standard GVHD prophylaxis with tacrolimus and methotrexate. All participants received standard antibiotic prophylaxis with levofloxacin and standard neutropenic fever treatment with intravenous cefepime (90%) or intravenous vancomycin along with intravenous aztreonam (10%). All antibiotic treatment was for prophylaxis or workup-negative neutropenic fever and discontinued at engraftment. The median total days of neutropenia was 7 (range: 5–14 days). No other antibacterial treatment was used for the entire duration of the study. Acyclovir was used for viral prophylaxis and fluconazole for fungal prophylaxis in all participants. None of the ten patients required treatment for viral or fungal infections while on study (Supplementary Table 1).

**Table 1:** Participants’ clinical characteristics

**Characteristic**	**Description**
*n*	10
Age (years) mean, median, range	55, 57, 52–62
Sex, male, female	6, 4
Body mass index in kg m^−^^2^, mean, median, range	27.24, 27.75, 22.4–31
Diagnosis	Three patients with myelodysplastic syndrome; two patients with acute myeloid leukemia; four patients with B acute lymphoblastic lymphoma; one patient with follicular lymphoma
Donor	HLA MRD T cell replete allogeneic HCT, 100%
Myeloablative conditioning	FluBu4, 90%; CyTBI, 10%
GVHD prophylaxis	Tacrolimus and methotrexate, 100%

FluBu4, fludarabine and busulfan myeloablative conditioning; CyTBI, cyclophosphamide and total body irradiation myeloablative conditioning.

Feasibility met the preset goal of 70% or greater adherence to scheduled dosages in 60% or more patients; eight out of ten patients (80%) received 70% or more of the scheduled doses. The median percentage dose taken by participants was 84% (Table 2). No adverse effects or toxicities attributed to RPS were observed. We recorded all GI adverse events occurring in the RPS cohort and the historical control cohort according to the Common Terminology Criteria for Adverse Events (CTCAE) v.5 (Supplementary Table 2). All these adverse events were deemed by treating clinicians unrelated to RPS, but were expected side effects of the conditioning chemotherapy patients received for their allogeneic HCT. All held RPS doses during the first 15 days after allogeneic HCT were due to expected transplant-related toxicities interfering with oral intake and not due to any study-related toxicities. Nausea and mucositis due to conditioning were the main allogeneic HCT side effects affecting oral intake where RPS had to be withheld. One patient developed biopsy-proven stage 1 acute GI GVHD with overall grade II acute GVHD (10%), demonstrating very low incidence in this small cohort. The University of Michigan Blood and Marrow Transplant Program’s Data and Safety Monitoring Board evaluated feasibility and recommended enrollment on phase 2.

**Table 2:** RPS administration and longitudinal specimen collection in ten patients with allogeneic HCT

Patient no.	1	2	3	4	5	6	7	8	9	10
Percentage of doses taken	78	99	97	33	88	79	31	74	92	97
Percentage of specimens collected	93	93	87	40	100	100	27	73	100	93

Taken together these results suggest that the use of RPS as a dietary intervention during allogeneic HCT is feasible, tolerable and safe.

### RPS promotes an increase in intestinal butyrate after allogeneic HCT

Stool specimens were collected from study participants at baseline before conditioning (day −7), nadir (approximately days 5–7), engraftment (approximately day 14) and day 100 (Fig. 1) using a collection device that allows for microbiome and metabolome analyses (OMNIgene container, Genotek). If patients were able to provide weekly samples, then stool was collected weekly. After processing, specimens were stored at −80 °C. The median percentage of stool specimens collected from each participant was 93% (Table 2).

**Fig. 2:**
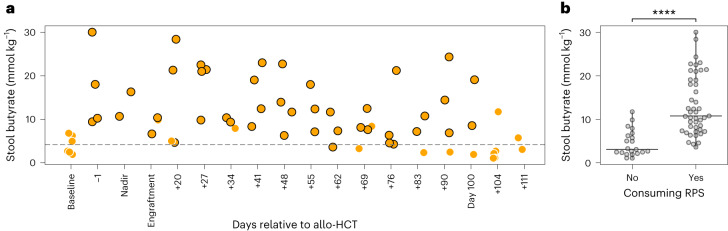
Stool butyrate over time in recipients of allogeneic HCT. **a**, Intention-to-treat analysis of stool butyrate levels in mmol kg^−1^ (*y* axis) as measured through allogeneic HCT where time (*x* axis) in days relative to allogeneic HCT is shown. Yellow dots with a black outline represent butyrate levels at time points when participants were consuming RPS; yellow dots with no outline represent butyrate levels at time points when participants were not taking RPS. **b**, Per protocol analysis of stool butyrate levels in mmol kg^−1^ (*y* axis) when participants were on RPS versus when they were not (*x* axis). A mixed random effects model was used to adjust for repeated measures from the same individuals. The median values for the no RPS and the yes RPS groups are 3.06 mmol kg^−1^ and 10.76 mmol kg^−1^, respectively. ****P value from the mixed random effects model coefficient for RPS versus stool butyrate level was 2 × 10^−13^.

To determine whether a regular and defined quantity of RPS impacted the host microbiome and its metabolites despite potential regular diet variation and standard mandated antibiotic use after HCT, we performed targeted metabolomic analyses on longitudinally collected stool specimens using HPLC to quantify the absolute amount of butyrate (Fig. 2a), acetate and propionate (Supplementary Fig. 1). We found that stool butyrate levels were significantly higher while participants were on RPS compared with when they were not on RPS at baseline and when RPS was held (median (interquartile range) = 10.76 mmol kg^−1^ (7.62–19.05) versus 3.06 (2.32–6.21), *P* < 0.0001, respectively) (Fig. 2b). We investigated whether the addition of intravenous antibiotics for neutropenic fever (besides the prophylactic antibacterial oral levofloxacin all patients were on) blunted the effect of RPS on stool butyrate levels. Only two participants recently received intravenous cefepime for workup-negative neutropenic fever at the time of engraftment. The stool butyrate level for the participant who was not taking RPS versus the participant who was taking RPS at that time point was 9.85 mmol kg^−1^ versus 6.63 mmol kg^−1^, respectively (Supplementary Table 3); however, these data are insufficient to draw any conclusions.

**Extended Data Fig. 1:**
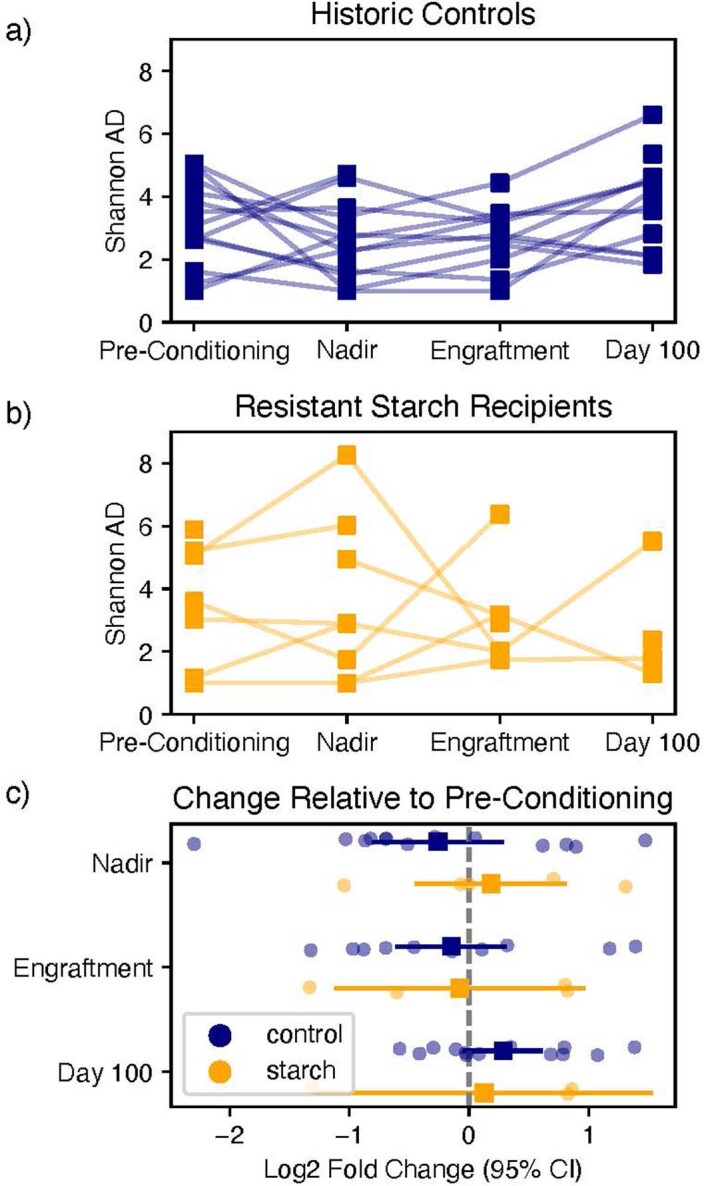
Shannon Alpha Diversity. **a)** Shannon Alpha Diversity in historic controls through allo-HCT. **b**) Shannon Alpha Diversity in RPS recipients through allo-HCT. **c**) Shannon Alpha Diversity relative to pre-conditioning comparing between historic controls (n = 15) and RPS recipients (n = 10) post allo-HCT. Values are Log2 fold change (log2) with mean and the 95% confidence interval of the mean Log2 fold change.

Given the changes in stool butyrate in response to RPS, we next set out to assess whether there were correlative changes in the microbiota. We performed 16S rRNA gene sequencing of stool microbiome DNA to determine the effects on microbial community structure at baseline in pre-HCT conditioning, and then compared it to changes at nadir, engraftment and day 100 so that the individual patient served as their own control. We further compared the intestinal microbes of recipients of RPS to historical controls undergoing allogeneic HCT at the same serial time points. The historical control patients were from the same center, underwent allogeneic HCT between 6 July 2016 and 23 June 2017, were similarly conditioned, and received similar immune prophylaxis and antibiotics (Supplementary Tables 1 and 4). As gut microbiome alpha diversity has been correlated with HCT outcomes, we first analyzed within each individual the overall microbial alpha diversity at each time point compared to the same participant’s baseline value (Extended Data Fig. 1a,b). While not statistically significant due to small numbers, overall recipients of RPS had preserved or increased alpha diversity, whereas historical controls tended to have reduced microbial diversity at nadir; however, by 3 months after allogeneic HCT, alpha diversity was increased (Extended Data Fig. 1c). Although we set out to collect stool samples weekly and a median of 93% of total samples was collected, not every patient was able to provide stool at every time point; hence, in Extended Data Fig. 1, we do not have data points for all ten patients at all four time points.

To determine the effect of RPS on diet–butyrate axis microbes, we analyzed microorganisms capable of producing butyrate based on a genetic and metagenomic survey of major known butyrate-producing pathways^37^ (Supplementary Fig. 2a). We also analyzed a specific set of microbes that comprised three bacterial species whose strains were implicated in RS degradation in healthy young adults: *Ruminococcus bromii*, *Bifidobacterium adolescentis* and *Bifidobacterium pseudocatenulatum*^34^ (Supplementary Fig. 2b). Overall, there were no statistically significant changes in these butyrate producers or RS degraders between the RPS and historic control cohorts. However, we observed a slight trend towards better preservation of previously reported butyrate-producing bacteria in the cohort consuming RPS compared to the historic controls, but with no change in the aforementioned RS degraders. These findings need to be validated in a larger sample.

Taken together, these results, consistent with previous reports, show that the process of allogeneic HCT disrupts the intestinal microbiome given the observed trend towards a decrease in microbial diversity at nadir (Extended Data Fig. 1) and in relative abundance of butyrate producers (Supplementary Fig. 2a). However, using RPS as a dietary intervention during the process of allogeneic HCT resulted in a significant increase in salutary intestinal SCFA butyrate levels as a by-product of microbial metabolism (Fig. 2).

### Exploratory analyses of plasma metabolites in allogeneic HCT

**Fig. 3:**
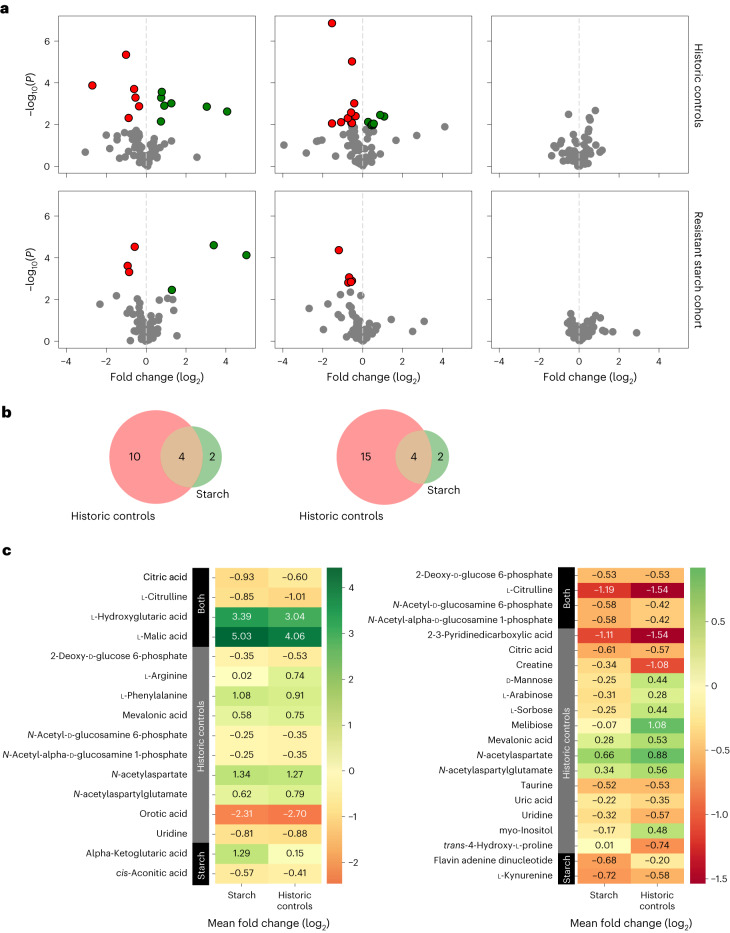
Plasma metabolites after allogeneic HCT compared to baseline. **a**, Volcano plots based on two-sided paired *t*-tests run by comparing each metabolite at different time points of the same patient within each cohort. The paired method was used to account for repeated sampling and a two-sided alternative hypothesis given the possibility of positive or negative correlations. For all plots, the *y* axis is the negative logarithm of the *P* value and the *x* axis is the logarithm of the fold change between the two time points being compared. The green and red dots show data points that are statistically significant. The top three plots are in the historical control cohort. Left, volcano plot comparing the nadir to baseline. Middle, engraftment compared to baseline. Right, day 100 compared to baseline. The bottom three plots are in the RS cohort. Left, volcano plot comparing the nadir to baseline. Middle, engraftment compared to baseline. Right, day 100 compared to baseline. These plots show changes in plasma metabolites at the nadir and engraftment after allogeneic HCT compared to baseline independent of whether recipients of allogeneic HCT received RPS. **b**, Venn diagram showing that overall, the RS cohort had far fewer metabolites than the historical control cohort, which were different at nadir compared to baseline (to the left) and engraftment compared to baseline (to the right). **c**, Heatmap table showing the specific metabolites that are different in each group (RS cohort, historical control cohort or both) at the nadir compared to baseline (to the left) and engraftment compared to baseline (to the right).

Given that RPS was associated with changes in intestinal butyrate levels, we next examined the potential effects of RPS on plasma metabolites by performing both targeted metabolomic analyses for SCFAs and global metabolomics for other metabolites (Supplementary Figs. 3 and 5 and Fig. 3). Blood specimens were collected using standardized protocols in EDTA tubes at baseline (day −7), nadir (approximately days 5–7), engraftment (approximately day 14) and day 100 (Fig. 1b). Samples were processed within 24 h to plasma and peripheral blood mononuclear cells (PBMCs). After processing, specimens were stored at −80 °C. One hundred percent of planned blood specimens were collected from each participant while on protocol.

**Extended Data Fig. 2:**
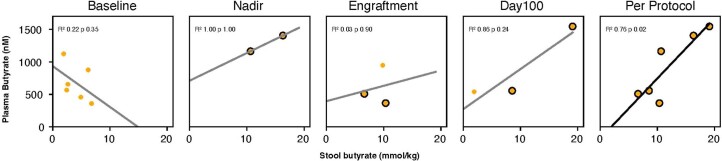
Plasma versus stool butyrate. Correlation graphs of plasma and stool butyrate in RPS recipients (n = 10) throughout allo-HCT. Only displaying timepoints at which observations of both stool and plasma metabolites were available. A Pearson’s correlation was completed for each time point, with a p-value determined based on a two-sided alternative hypothesis given the possibility for negative correlation and against the exact distribution of r.

We longitudinally assessed the potential effect of RPS on plasma concentrations of SCFAs using mass spectrometry (MS). We found no significant differences in butyrate, propionate and acetate across all time points and between recipients of RPS and historical controls after allogeneic HCT (Supplementary Fig. 3) with the exception of a bimodal state at day 100 in butyrate concentrations in recipients of RPS (Supplementary Fig. 3a). Although butyrate is rarely detected in plasma, but was increased in the stool of patients on RPS, we also compared and correlated plasma versus stool SCFAs in recipients of RPS (Extended Data Fig. 2 and Supplementary Fig. 4) and found a significant correlation between stool and plasma butyrate concentrations in recipients of RPS (*P* = 0.02) (Extended Data Fig. 2). Given that stool samples were not always available, we have few data points at each time point where both plasma and stool butyrate levels were available and cannot draw conclusions regarding the correlation between plasma and stool butyrate levels at each time point.

**Fig. 4:**
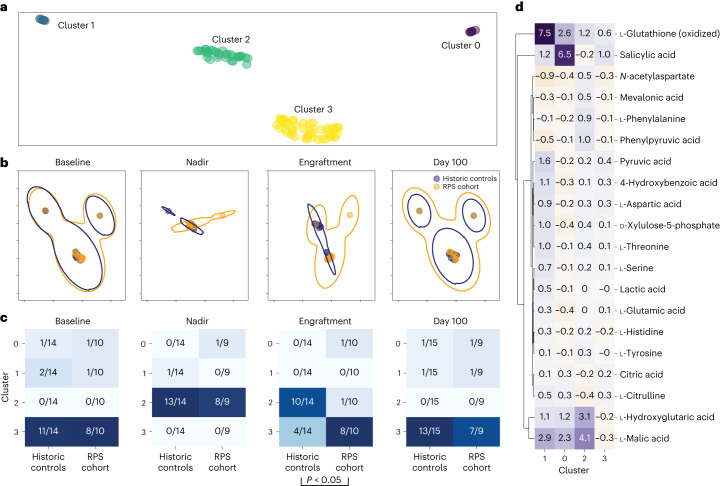
Plasma metabolites in historic controls versus recipients of RPS. **a**, UMAP ordination of median-centered plasma metabolite levels revealed four distinct clusters of metabolites. **b**, UMAP ordination stratified according to clinical group (historic controls in blue or recipients of RPS in orange), with the largest divergence in overlap at engraftment. **c**, Contingency tables of plasma metabolite groups stratified according to clinical group (historical controls or recipients of RPS) at four time points with baseline to the far left, followed by nadir, then by engraftment and then by day 100 to the far right. Chi-squared contingency testing *P* values are reported, with only significant differences between the RPS and historical control cohorts at engraftment. **d**, Regression revealed a subset of metabolites driving each cluster, with cluster 3 (dominant at baseline) notable for metabolite levels close to the median.

We next determined the effect of RPS on all other plasma metabolites (more than 200 metabolites) in an unbiased manner using global metabolomic analyses. Eighty-two metabolites showed discrete peaks. Major changes in global plasma metabolites were observed at the time points after allogeneic HCT compared to baseline within each of the patients in the non-RPS and RPS recipient groups (Supplementary Fig. 5). Longitudinally significant changes in plasma metabolites occurred when comparing the nadir and engraftment time points to baseline, but not at day 100, consistent with the changes seen in the microbiota (*P* < 0.0001) (Fig. 3). To compare more than 200 plasma metabolites between the historical control and RPS cohorts, we performed an ordination and cluster-based analysis of plasma metabolites where we ordinated all metabolites into two dimensions using uniform manifold approximation and projection (UMAP) and clustered all specimens into four clear clusters using hierarchical density-based spatial clustering of applications with noise (HDBSCAN) (Fig. 4a). When we applied the meta-data to the ordinated specimens to compare the historical control and RPS cohorts at each time point, we found that at baseline specimens were very similar between the historical control and RPS groups indicating no substantial metabolic differences between the groups before HCT and RPS intervention. However, differences clearly emerged between the two cohorts at nadir and engraftment, although these differences were less apparent by day 100 (Fig. 4b).

Using the clusters with the meta-data, we further asked if there were specific clusters enriched in the RPS versus the historical control group when comparing the time points after allogeneic HCT to baseline. We found that at baseline the distribution by cluster was similar as expected, and that at nadir both groups showed cluster changes. However, the RPS group metabolome was more stable over time, whereas the historic control group had more changes over time, with statistically significant differences between the two groups at engraftment (*P* < 0.01) (Fig. 4c). We then examined the specific metabolites that define each cluster and found that cluster 3, which is dominant in both cohorts at baseline and only in the RPS cohort at engraftment after allogeneic HCT, is notable for a lack of overrepresented metabolites and is mostly even across the board when comparing to other clusters (Fig. 4d).

Taken together, these results show that the process of allogeneic HCT alters plasma metabolites compared to baseline and that these alterations start to resolve by 3 months after allogeneic HCT; however, the resolution may be expedited and the disruption mitigated in the RPS cohort compared with controls. Thus, using RPS as a dietary intervention during allogeneic HCT may stabilize plasma metabolic changes after allogeneic HCT.

## Discussion

In this prospective interventional study, we determined that administration of commonly available RPS as a dietary prebiotic in recipients of allogeneic HCT is clinically feasible, tolerated by patients and may mitigate microbiome and metabolite disruption after allogeneic HCT. We measured the feasibility of administering RPS to human recipients of allogeneic HCT by presetting a goal of 70% or greater adherence to scheduled dosages in 60% or more patients; this was met as eight out of ten patients (80%) received 70% or more of the scheduled doses. We measured the microbial SCFA butyrate, which has been shown to promote salutary effects after allogeneic HCT in mice, and found that it was significantly increased in the stool of recipients of RPS at multiple time points after allogeneic HCT when patients were on RPS.

We examined the changes in microbiome structure, specifically the RS degraders and butyrate producers given the significant increase observed in stool butyrate when patients were on RPS. We focused on the specific RPS diet–stool butyrate axis microbes based on previous reports from healthy cohorts^34,37^. Compared with the previous healthy cohort datasets, more than 98% of the sequences derived from the allogeneic HCT samples (both treatment and historic controls) were identical to amplicon sequence variants (ASVs) identified in the healthy cohort. These data suggest that at least at this level of discrimination, the microbes present in the allogeneic HCT cohort are a subset of microbes found in healthy individuals. The two populations of RPS degrader bacteria that bloom in patients with allogeneic HCT consuming RPS were also noted in the healthy cohort study after RPS (Supplementary Fig. 6)^34^. However, the Bifidobacteria populations that bloomed in the healthy cohort on RPS were not observed in the allogeneic HCT cohort on RPS. Like the healthy cohort data on RPS, butyrate producers also showed no meaningful difference in the relative abundance or richness between the control and treatment groups of allogeneic HCT. These data suggest that the impact of RPS in causing an increase in butyrate is observed in both allogeneic HCT and healthy cohorts, with similar change in some but not all of the bacteria that are RPS degraders. Nonetheless, these comparisons ought to be interpreted with caution across the different studies and patient populations. It is also important to note that the sample collection demonstrated variability due to expected variation in provision of stool samples on a specific day by each of the patients, which is reflected in the stool butyrate analyses from individual patients across multiple time points (Supplementary Fig. 7). However, despite some missed time points, aggregate data were consistent with significant change in butyrate levels (Fig. 2b). Furthermore, consistent with previous reports, our data demonstrated a trend towards loss of diversity after allogeneic HCT, but this may be mitigated by RPS administration by maintaining a more stable equilibrium of butyrogenic bacteria resulting in the observed significant increase in salutary intestinal SCFA butyrate levels as a by-product of microbial metabolism.

In exploratory analyses, we measured plasma metabolites at baseline and multiple time points after allogeneic HCT in historical controls and recipients of RPS and found that the composition of plasma metabolites changed after allogeneic HCT compared to the individual patient’s baseline. These changes were most pronounced at nadir after allogeneic HCT and normalized by 3 months after allogeneic HCT. We used a new approach of clustering and ordination-based analysis for human plasma metabolomics data to compare plasma metabolites after allogeneic HCT in the RPS and historical control cohorts. Our rationale for using this approach was based on the observation that plasma metabolites were not independent from one another, but rather they were highly correlated. Thus, we hypothesized that plasma metabolite levels may fall into identifiable discrete clusters as shown in Fig. 4a. The clustering and ordination-based analysis revealed that the cluster most dominant at baseline was notable for metabolite levels close to the median and that the RPS cohort had more plasma metabolites after allogeneic HCT in a similar state to that observed at baseline compared to the historic controls. In summary, the changes in plasma metabolites may be more stable after allogeneic HCT in the RPS group compared to the allogeneic HCT control group, which may be indicative of a stable equilibrium of production and consumption by intestinal microbes.

Several studies showed that the prebiotics inulin and fructo-oligosaccharides (FOS) increased intestinal microbiota diversity and decreased disease activity in patients with inflammatory bowel disease (IBD)^38,39^. However, there is lack of sufficient prospective clinical data on the role of prebiotics in human recipients of allogeneic HCT, and whether it can impact microbiome structure and metabolites^40^. A recent study tested the prebiotic FOS in recipients of allogeneic HCT^41^. This phase 1 trial found that FOS were safe and tolerable when administered at 10 g per day for 21 days in recipients of allogeneic HCT. However, the effects of FOS on the intestinal microbiota and its metabolites with this trial design were fleeting as FOS administration was not associated with any changes in SCFAs in that study. By contrast, our data demonstrate feasibility and prolonged change in microbiome structure and metabolome. This longitudinal impact on the SCFA, butyrate, when taken in light of and compared to other studies exploring the impact of different RS, highlights that RPS may be the more rational and efficient way to change the structure of the intestinal microbiome and metabolites compared to other RS. However, other differences must also be noted, including study design. Specifically, our study design allowed for longer administration of the dietary intervention, which in turn probably translated into more lasting effects on the intestinal microbiota and metabolites after allogeneic HCT. Second, we administered RPS for the first 100 days after allogeneic HCT because during this time there are multiple known risk factors that can negatively alter the microbiome, such as conditioning regimens, antibiotics and inflammation. We also designed the timing of RPS administration, keeping in mind tolerability in this patient population who experience many GI side effects from their conditioning regimens. The rationale for initiating RPS on day −7 is to precede initiation of myeloablative conditioning because tissue injury ensues during or immediately after conditioning^42^. While there are many potential sources for RPS, we chose RPS produced by Bob’s Red Mill because it is easily commercially available, economical and previously well tolerated in healthy volunteers^33^. However, we treated and administered RPS by Bob’s Red Mill as a medication to allow for stringent and rational quantifiable administration under research pharmacy and on an investigational new drug (IND) application from the U.S. Food and Drug Administration (FDA).

Allogeneic HCT and therapies after it are complex, widely variable processes, including but not limited to conditioning regimen, antibiotic administration, donor source and immunosuppressive prophylaxis, which can all have effects on the microbiome^43,44^. We attempted to standardize most of these factors in our study participants and in those we used as a control cohort. With regards to the donor source, while the donors for the RPS cohort were related and those for the historical controls were unrelated, all donors for both cohorts were fully HLA-matched and T cell replete. While the degree of HLA matching and presence or absence of T cells in the donor inoculum are the more important factors with regard to the effects on tissue injury and microbial dysbiosis after allogeneic HCT, our data comparing the two groups of patients must be interpreted within the limitations of matched related versus matched unrelated donors along with the caveats that are germane to any comparisons across studies. However, it is also important to note that the increase in butyrate from before RPS to after RPS was observed in most patients (Supplementary Fig. 7) despite the complex therapies any individual patient typically receives in the context of allogeneic HCT.

Most studies that examined the microbiome and its metabolites in recipients of allogeneic HCT focused on a single time point after allogeneic HCT, particularly at engraftment. In our study, we performed longitudinal, correlative analyses that allowed us to use each patient’s baseline as a reference. Only one of ten patients developed biopsy-proven, stage 1 acute GI GVHD with overall grade II acute GVHD demonstrating very low incidence, although the sample size was small.

Our study has limitations. A major limitation is the small number of participants, particularly to determine the impact of an intervention on a clinical end point, such as acute GVHD. A larger cohort of patients is currently being enrolled to determine the clinical impact on acute GVHD. Another limitation is that the RPS diet–stool butyrate axis microbes examined in our study are based on previous reports from healthy cohorts^34,37^ rather than from patients with allogeneic HCT. Future metagenomic studies of butyrate-producing and RS degradation pathways in patients with allogeneic HCT are needed. Another limitation is that the donor source for recipients of RPS was from related donors, whereas that for historical controls was from unrelated donors; however, HLA matching and presence of T cells, the most important donor factors expected to affect the microbiome, were the same for both groups. The second phase of this study, which is currently underway, will include a randomized cohort receiving placebo who will serve as contemporaneous controls from the same study.

Our study provides preliminary data suggesting that a dietary intervention using RPS altered the intestinal microbial metabolite SCFA, butyrate, and was feasible and safe in recipients of allogeneic HCT. These data demonstrate proof of principle for RPS as a feasible prebiotic intervention in recipients of allogeneic HCT that may moderate disruption of microbiome-derived metabolites, and sets the stage for further exploration of RPS and its effects on the microbiome in human disease.

## Methods

### Study participants

From 26 April 2017 to 30 September 2018, ten adults aged 52–62 years were recruited at the University of Michigan. This prospective feasibility single-center clinical study was done under FDA IND no. 132208. Participants were fully informed of the study and signed the consent form before any study procedures. The trial was reviewed by the University of Michigan Protocol Review Committee and was approved by the institutional review board. The Rogel Cancer Center’s and the University of Michigan Blood and Marrow Transplant Program’s DSMBs monitored the study quarterly. The study protocol specified comparing study patient samples to samples at identical time points of patients who were not on the study as external historical controls. Historical controls received allogeneic HCT at the University of Michigan from 6 July 2016 to 23 June 2017.

### Inclusion criteria

Patients undergoing HLA MRD myeloablative allogeneic HCT were included in the study.

### Exclusion criteria

These included IBD, a history of gastric bypass surgery, active *Clostridium difficile* infection, active participation in an alternative GVHD prevention trial, and any physical or psychological condition that, in the opinion of the investigator, would pose unacceptable risk to the patient or raise concern that the patient would not comply with the protocol procedures.

### Feasibility goal

This trial had a preset feasibility goal of 70% or greater adherence to scheduled dosages in 60% or more patients.

### Stool and blood specimen collection

Stool specimens were collected from the study participants at baseline before conditioning (day −7), nadir (approximately days 5–7), engraftment (approximately day 14) and day 100 using a collection device that allows for microbiome and metabolome analyses (OMNIgene container, Genotek). After processing, stool specimens were stored at −80 °C. If patients were able to provide weekly samples, then stool was collected and analyzed weekly; however, stool was also collected at the four aforementioned time points which were prespecified and required for the blood specimens: baseline; nadir; engraftment; and day 100. Blood specimens were collected using standardized protocols in EDTA tubes at baseline (day −7), nadir (approximately days 5–7), engraftment (approximately day 14) and day 100. Samples were processed within 24 h to plasma and PBMCs. After processing, blood specimens were stored at −80 °C.

### Feasibility analysis

Feasibility was evaluated using the preset goal of 70% or greater adherence to scheduled dosages in 60% or more patients and exceeded the preset goal as eight out of ten patients (80%) received 70% or more of the scheduled doses.

### 16S rRNA gene variable region amplicon data analysis

Reads were generated from specimens according to the routine protocol of the University of Michigan Microbiome Core. They were amplified with Earth Microbiome Project primers targeting the V4 variable region of the gene (intended to generate full overlap in the paired-end reads) with an error-correcting polymerase, followed by sequencing with the Illumina MiSeq platform. Processing of the raw FASTQ files was as per Golob et al.^44^ and Minot et al.^45^ 2022: ASVs were generated with DADA2. ASVs were placed onto a de novo phylogenetic tree (generated with RAxML) of full-length 16S rRNA alleles with pplacer. Alpha diversity metrics were derived from the placed ASVs using guppy. Taxonomic classifications for each ASV were derived by EPA-ng from the phylogenetic placements. Butyrate generators were identified as in Vital et al.^37^. RS degraders were identified based on the reported organisms in Baxter et al.^34^.

### SCFA analysis

The SCFA standard mixture was obtained from Sigma-Aldrich (catalog no. CRM46975). A ^13^C-SCFA stool mixture (catalog no. SBR00035-1ML, Sigma-Aldrich) was used as the internal standard. Analytical reagent-grade 3-nitrophenylhydrazine (3NPH) HCl (catalog no. N21804), EDAC HCl (catalog no. 341006), HPLC-grade pyridine (catalog no. 270407), liquid chromatography (LC)–MS-grade acetonitrile (catalog no. 34851), water (catalog no. 270733) and formic acid (catalog no. 5438040450) were also purchased from Sigma-Aldrich. The working standard solutions were created by performing serial dilution from the 10 mM stock solution down to the nM range using freshly prepared 50% (v/v) aqueous acetonitrile in water. The chemical derivatization protocol was modified from Han et al.^46^. Briefly, 20 µl of the working standard solutions or samples was mixed with 40 µl of 200 mM 3NPH in 50% aqueous acetonitrile, 120 mM EDAC 6% (v/v) pyridine solution in the same solvent and 4 µl of the internal standard in a Verex glass vial. The mixture was reacted at 40 °C for 30 min. After the reaction, 96 µl of 0.1% formic acid in 10% acetonitrile solution was added to the mixture to quench the reaction. Then, 30 µl of the reaction solution was transferred to a new HPLC vial and a 2-µl aliquot of each solution was injected into the LC–tandem MS (MS/MS) instrument. Each modified SCFA was optimized in Agilent MS for detection through an Agilent Optimizer 2.0. All optimized SCFA information was combined and an LC–multiple reaction monitoring (MRM) was created. Retention time for each SCFA was determined from two transitions. Then, the MRM method was transformed into a dynamic MRM with all the retention time and MS information for the final LC–MS acquisition.

Our LC–MS/MS metabolomics analysis was performed as described previously^47^. Briefly, the Agilent Technologies Triple Quad (QQQ) 6470 LC–MS system, which consisted of a 1290 Infinity II LC Flexible Pump (Quaternary Pump), 1290 Infinity II Multisampler, 1290 Infinity II Multicolumn Thermostat with 6 port valve and 6470 Triple Quad mass spectrometer, was used for label-free targeted metabolomics analysis. Retention time for each metabolite was measured from a pure standard solution or a mixed standard solution. The LC–MS/MS methods were created with dynamic MRM with retention times, retention time windows and transitions of all standard compounds.

A Waters Acquity UPLC BEH TSS C18 column (2.1 × 100 mm, 1.7 µm) column was used with mobile phase A, consisting of 0.1% formic acid in water, and mobile phase B, consisting of 0.1% formic acid in acetonitrile. The gradient program was as follows: mobile phase B was held at 15% for 1 min, increased to 55% for 19 min, then to 99% for 20 min and held for 2 min before going to the initial condition and held for 4 min. The column was held at 40 °C; 2 µl of sample was injected into the LC–MS with a flow rate of 0.3 ml min^−1^. Calibration of the 6470 Triple Quad mass spectrometer was achieved using the Agilent ESI-low Concentration Tuning Mix. The key parameters of electrospray ionization were: gas temperature 300 °C; gas flow at 5 l min^−1^; nebulizer at 45 psi; sheath gas heater at 250 °C; sheath gas flow at 11 l min^−1^; capillary at 3,500 V; and delta EMV at 200 V. The dynamic MRM scan type was used with a 0.07 min peak width. A delta retention time of ±1 min, a fragmentor of 40 eV and a cell accelerator of 5 eV were incorporated in the method.

### Plasma metabolomics analysis

Plasma metabolomics was performed with the Agilent ZORBAX Rapid Resolution High Definition Extend-C18 (2.1 × 150 mm, 1.8 µm) and ZORBAX Extend Fast Guards for UHPLC. Ion paring (IP) stock solution was prepared by mixing GC-grade 450 ml methanol, 35.8 ml tributylamine (TBA) and 12.9 ml acetic acid. Solution A was prepared with 1,000 ml of HPLC-grade water, 1,034 µl deactivator and 34.25 ml IP stock solution. Solution B was prepared with 1,000 ml of HPLC-grade methanol, 1,034 µl deactivator and 34.25 ml IP stock solution. Solvent A was 97% water and 3% methanol with 15 mM acetic acid and 10 mM TBA at a pH of 5. Solvent C was 15 mM acetic acid and 10 mM TBA in methanol. The washing solvent D was acetonitrile. The LC system seal washing solvent was 90% water and 10% isopropanol. The needle washing solvent was 50% methanol and 50% water. The LC gradient profile was: at 0.25 ml min^−1^, 0–2.5 min, 100% A; 7.5 min, 80% A and 20% C; 13 min, 55% A and 45% C; 20 min, 1% A and 99% C; 24 min, 1% A and 99% C; 24.05 min, 1% A and 99% D; 27 min, 1% A and 99% D; at 0.8 ml min^−1^, 27.5–31.35 min, 1% A and 99% D; at 0.6 ml min^−1^, 31.50 min, 1% A and 99% D; at 0.4 ml min^−1^, 32.25–39.9 min, 100% A; at 0.25 ml min^−1^, 40 min, 100% A. The column temperature was kept at 35 °C, samples were kept at 4 °C and the injection volume was 2 µl. The 6470 Triple Quad mass spectrometer was calibrated with the ESI-low Concentration Tuning Mix. The source parameters were: gas temperature 150 °C; gas flow 10 l min^−1^; nebulizer 45 psi; sheath gas heater at 325 °C; sheath gas flow at 12 l min^−1^; capillary at −2,000 V; and delta EMV at −200 V. A dynamic MRM scan was used with a 0.07 min peak width; acquisition time was 24 min. A delta retention time of ±1 min, a fragmentor of 40 eV and a cell accelerator of 5 eV were incorporated in the method. Raw EV values were then median-centered per compound (each raw EV value was divided by the median value for that compound).

### Statistical analysis

We performed an intention-to-treat analysis of stool SCFA (butyrate, propionate and acetate) levels in mmol kg^−1^ as measured through allogeneic HCT where time was days relative to allogeneic HCT. We also performed a per protocol analysis of stool SCFA (butyrate, propionate and acetate) levels in mmol kg^−1^ when participants were on RPS versus when they were not. A mixed random effects model was used to adjust for repeated measures from the same individuals.

We generated volcano plots based on a Student’s *t*-test, showing changes in plasma metabolites at the after allogeneic HCT time points compared to baseline within each patient. We also generated volcano plots based on paired *t*-tests, run by comparing each metabolite at different time points of the same patient within each cohort. For all plots, the *y* axis was the negative logarithm of the *P* value and the *x* axis was the logarithm of the fold change between the two time points being compared.

UMAP ordination (the umap-learn library in Python) of the specimens was generated after calculating the cosine distance between specimens based on the median-centered plasma metabolite data, specifying ten nearest neighbors. The result was four distinct clusters that could be readily identified by HDBSCAN (minimum sample of 1 and minimum cluster size of 5). Generalized linear modeling was used to determine the compounds most associated with at least one cluster. The number of specimens per cluster was established for each time point and each cohort.

Python v.3.9 with umap-learn, statmodels and scikit-learn libraries were used for the data analysis, with all parameters noted in the Methods and text (defaults were used otherwise). Visualizations were accomplished using the matplotlib and seaborn libraries.

### Reporting summary

Further information on research design is available in the Nature Portfolio Reporting Summary linked to this article.

## Online content

Any methods, additional references, Nature Portfolio reporting summaries, source data, extended data, supplementary information, acknowledgements, peer review information; details of author contributions and competing interests; and statements of data and code availability are available at 10.1038/s41591-023-02587-y.

### Supplementary information

Supplementary Information Supplementary Figs. 1–8, Appendix A (Clustering and ordination-based analysis of human plasma metabolomics data), Appendix B (Butyrate taxons), Appendix C (List of metabolites in global snapshot metabolomics) and Protocol.

Reporting Summary

Supplementary Table 1 Supplementary Tables 1–4.

## Data Availability

Data in this study have been presented, where possible, in aggregated form. All metabolite data generated in the preparation of this study are available at 10.5281/zenodo.8015340. All microbiome data (16S rRNA amplicons) generated during the preparation of this study have been deposited in the NCBI Sequence Read Archive with BioProject ID PRJNA953087. This study was prospectively registered. The ClinicalTrials.gov registration is NCT02763033. The study is also under FDA IND application no. 132208. The ten patients from the pilot feasibility study presented in this study represent the initial part of a phase 2 randomized trial currently enrolling 60 additional patients to determine the clinical impact on acute GVHD.
