# Supplementary material for: Feasibility of a dietary intervention to modify gut microbial metabolism in patients with hematopoietic stem cell transplantation
Source: Nat Med. 2023 Oct 19;29(11):2805–13. doi: 10.1038/s41591-023-02587-y (PMC10667101; doi:10.1038/s41591-023-02587-y)
Supplement: Supplementary file 1 — Supplementary Figs. 1–8, Appendix A (Clustering and ordination-based analysis of human plasma metabolomics data), Appendix B (Butyrate taxons), Appendix C (List of metabolites in global snapshot metabolomics) and Protocol. [file 41591_2023_2587_MOESM1_ESM.pdf]

nature medicine

Article

https://doi.org/10.1038/s41591-023-02587-y

Feasibility of a dietary intervention to modify gut microbial metabolism in patients with hematopoietic stem cell transplantation

In the format provided by the authors and unedited

a) Supplemental Figure 1

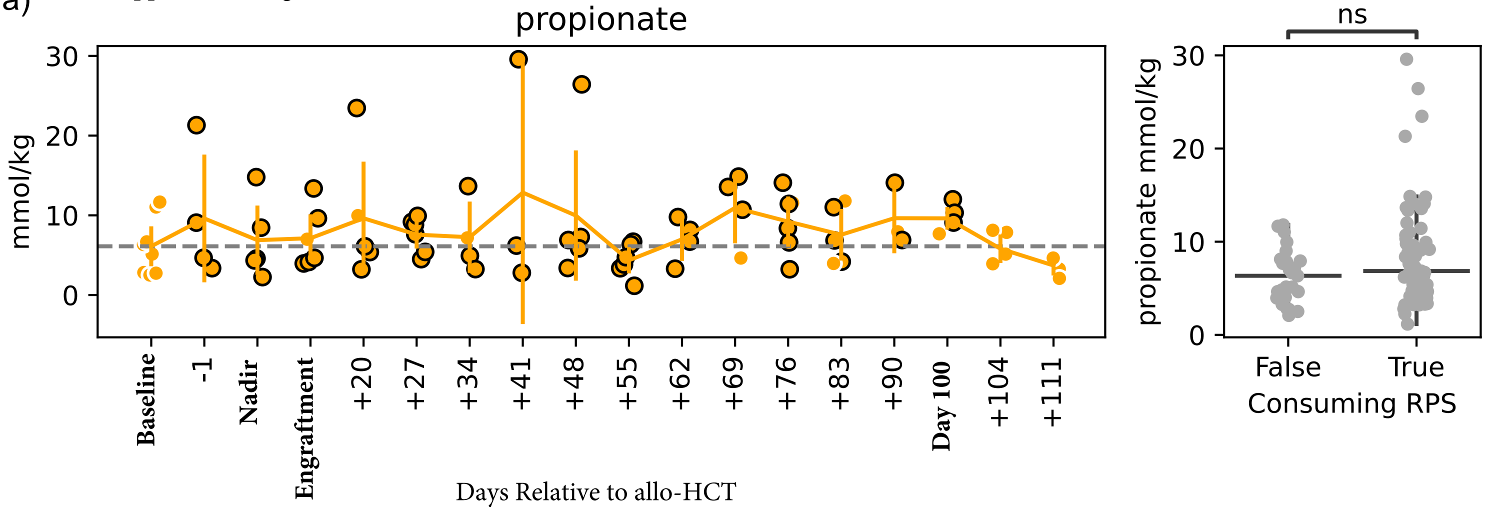

Supplemental Figure 1

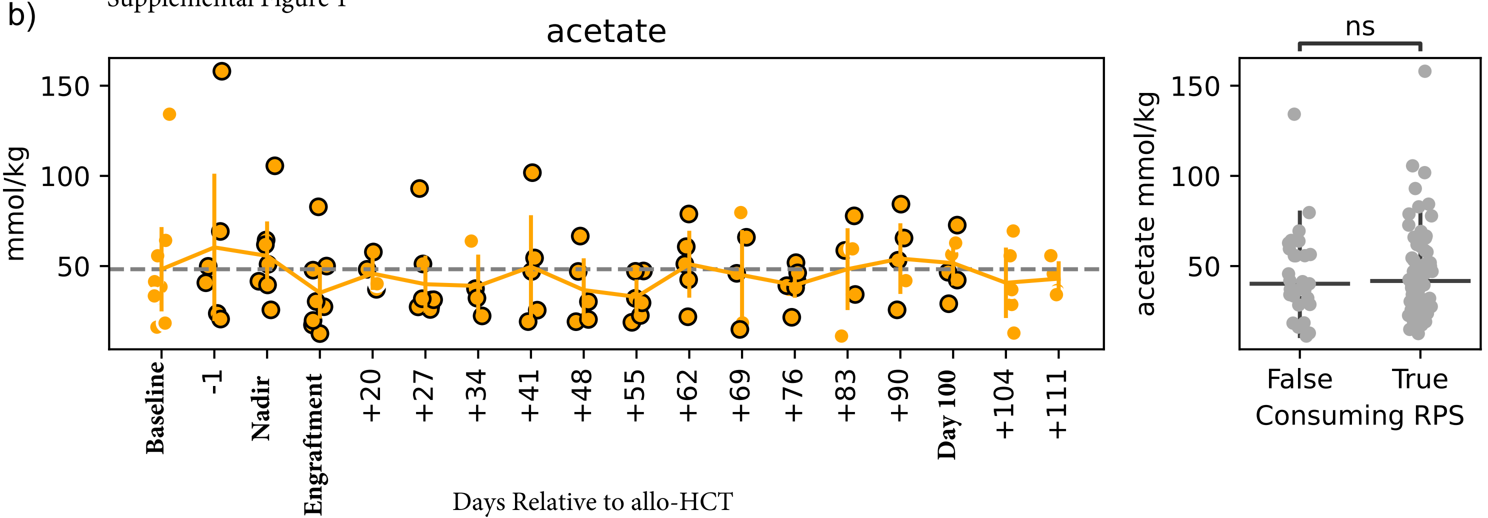

Supplemental Figure 2

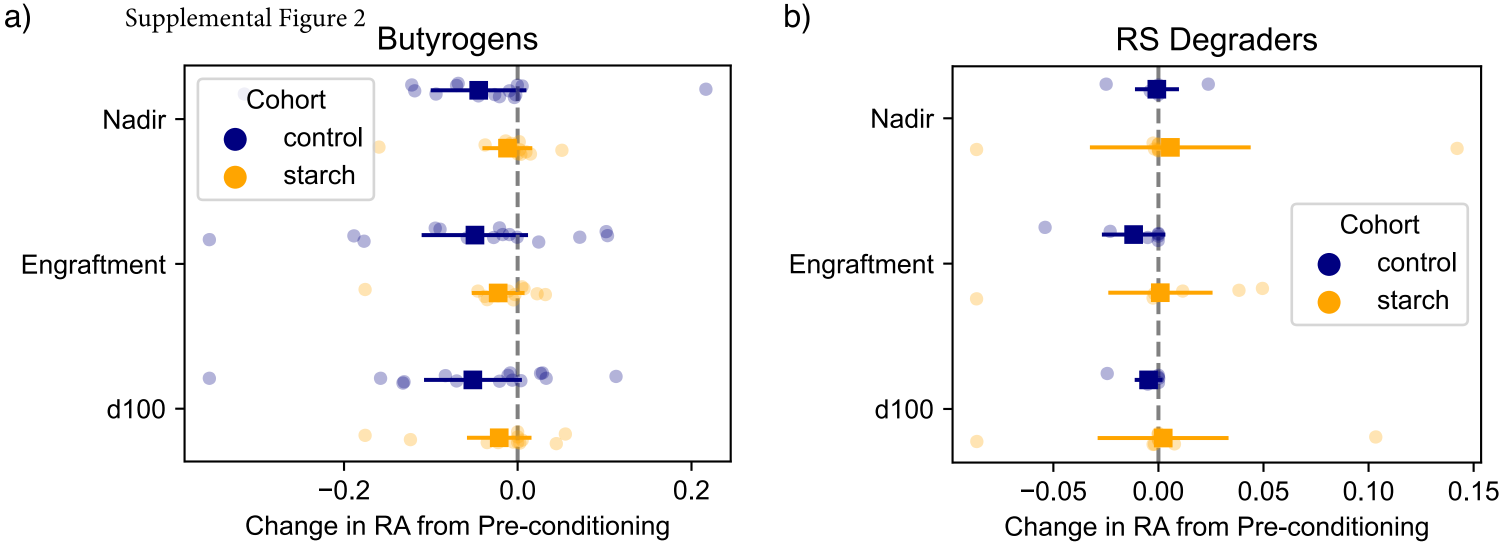

Supplemental Figure 3

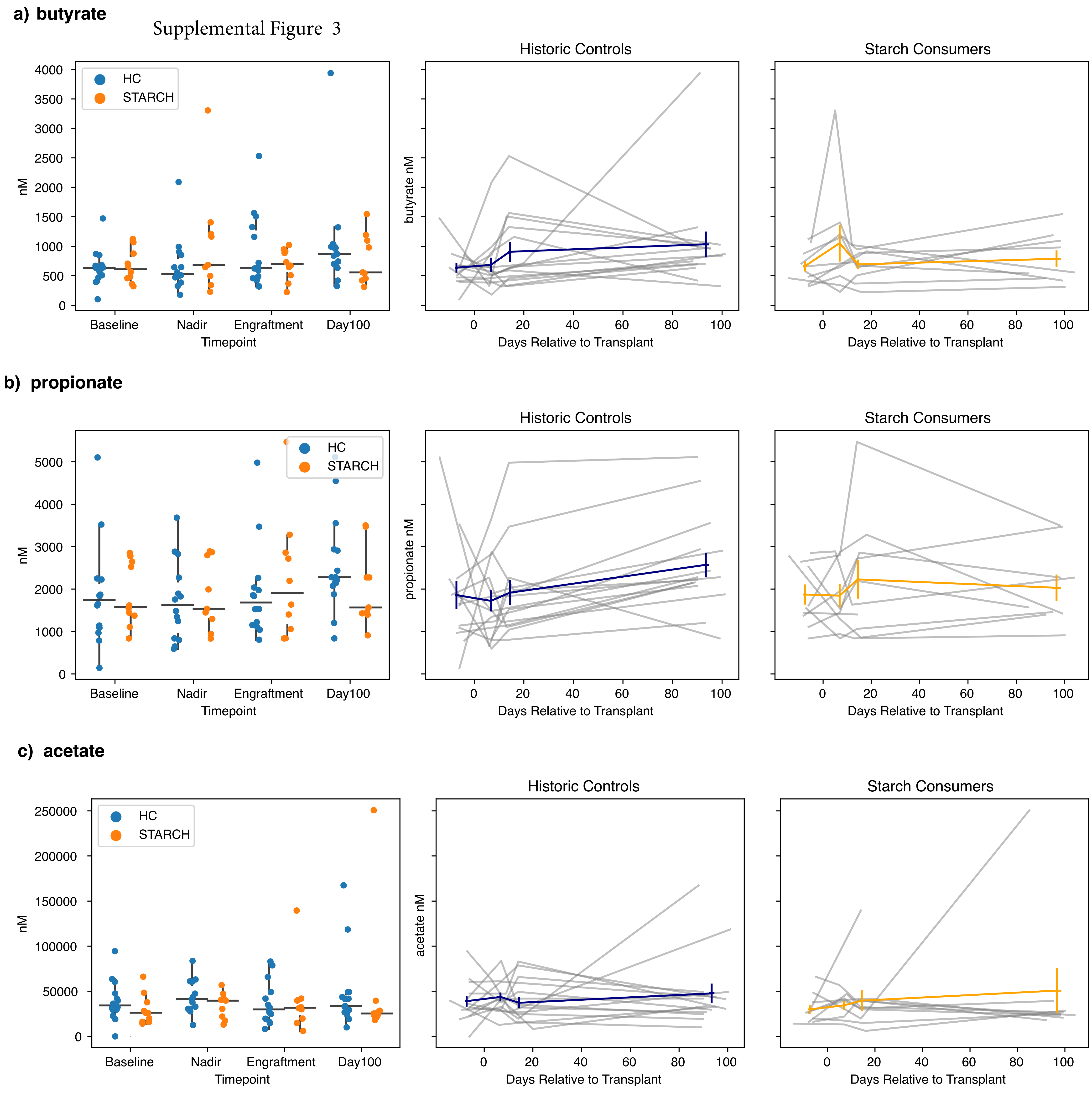

Supplemental Figure 4

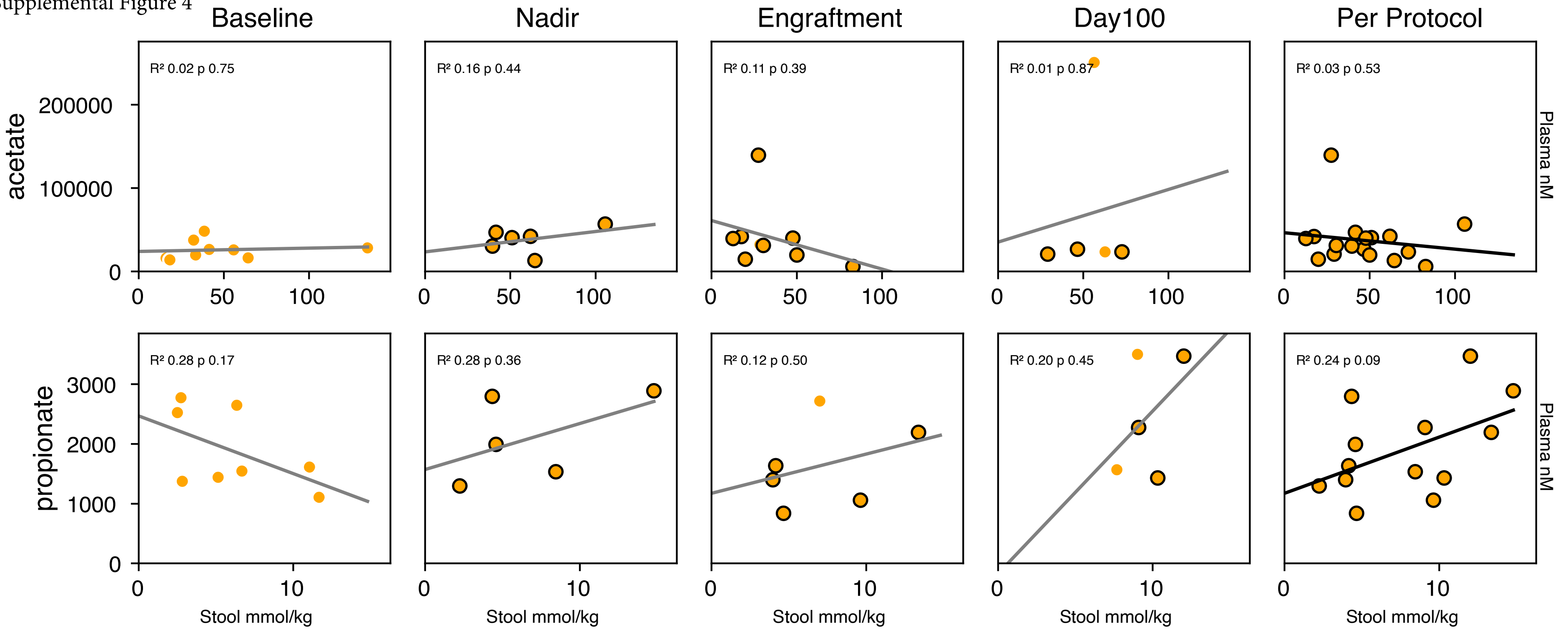

Supplemental Figure 5

HC group

Nadir vs Baseline

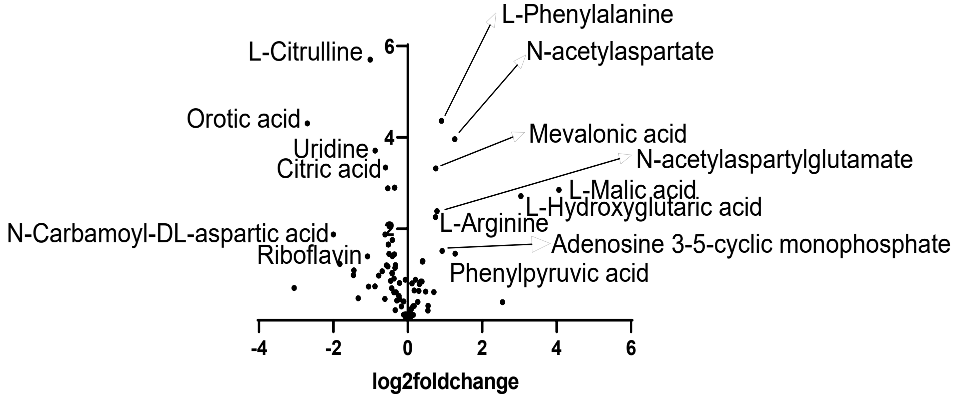

Engraftment vs Baseline

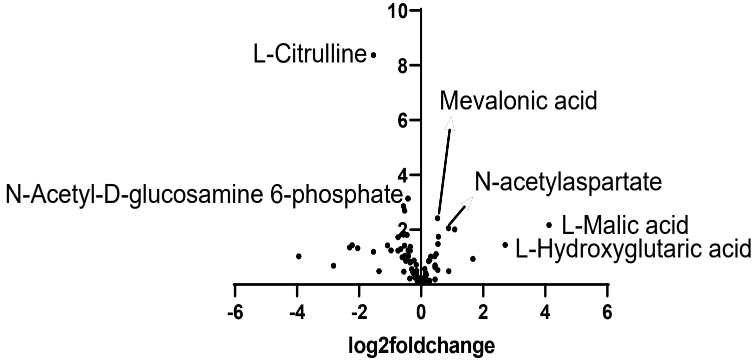

Day100 vs Base

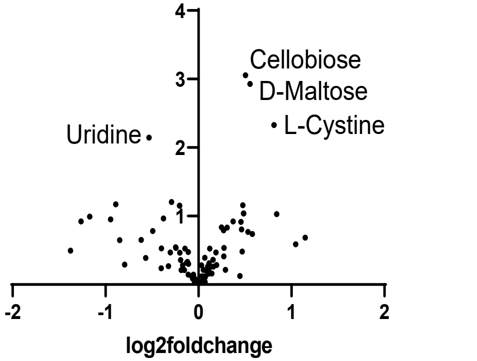

Starch group

Nadir vs Baseline

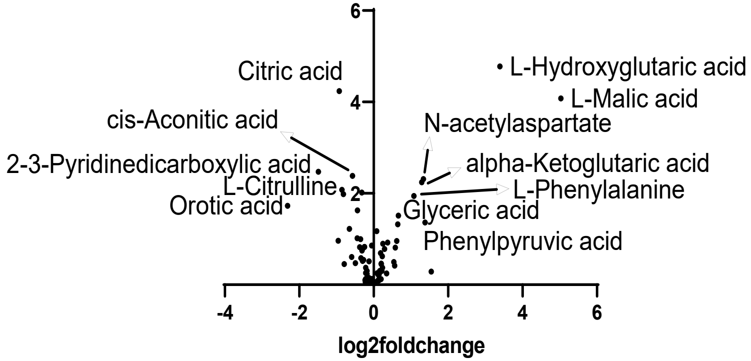

Engraftment vs Baseline

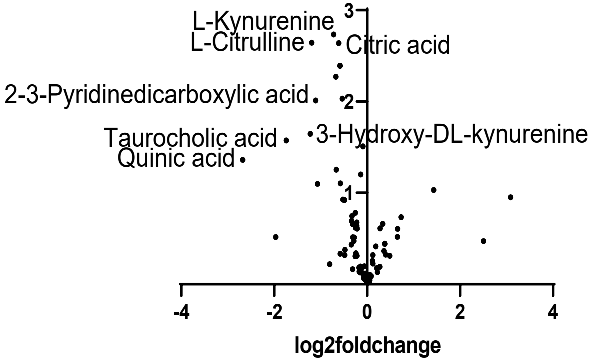

Day100 vs Base

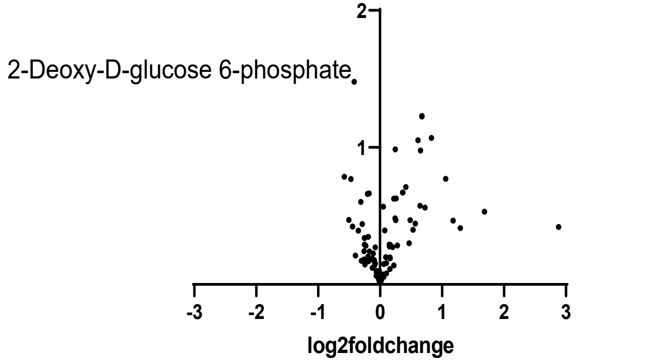

Supplemental Figure 6

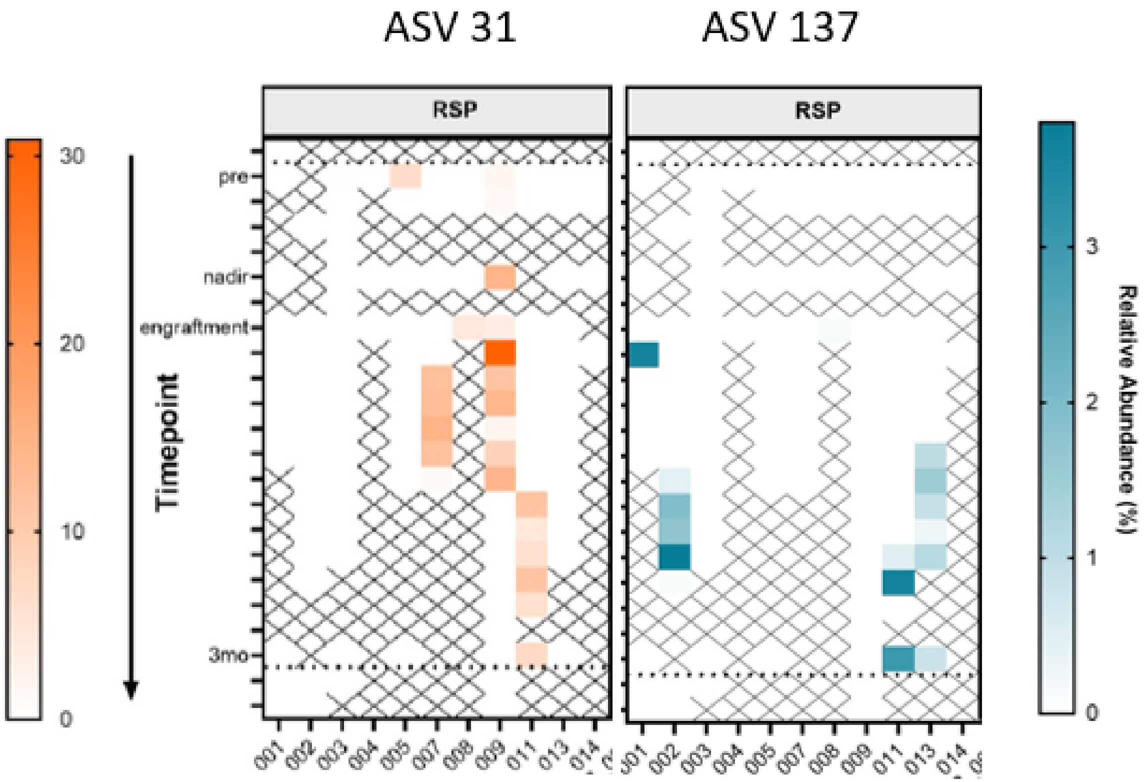

Supplemental Figure 7

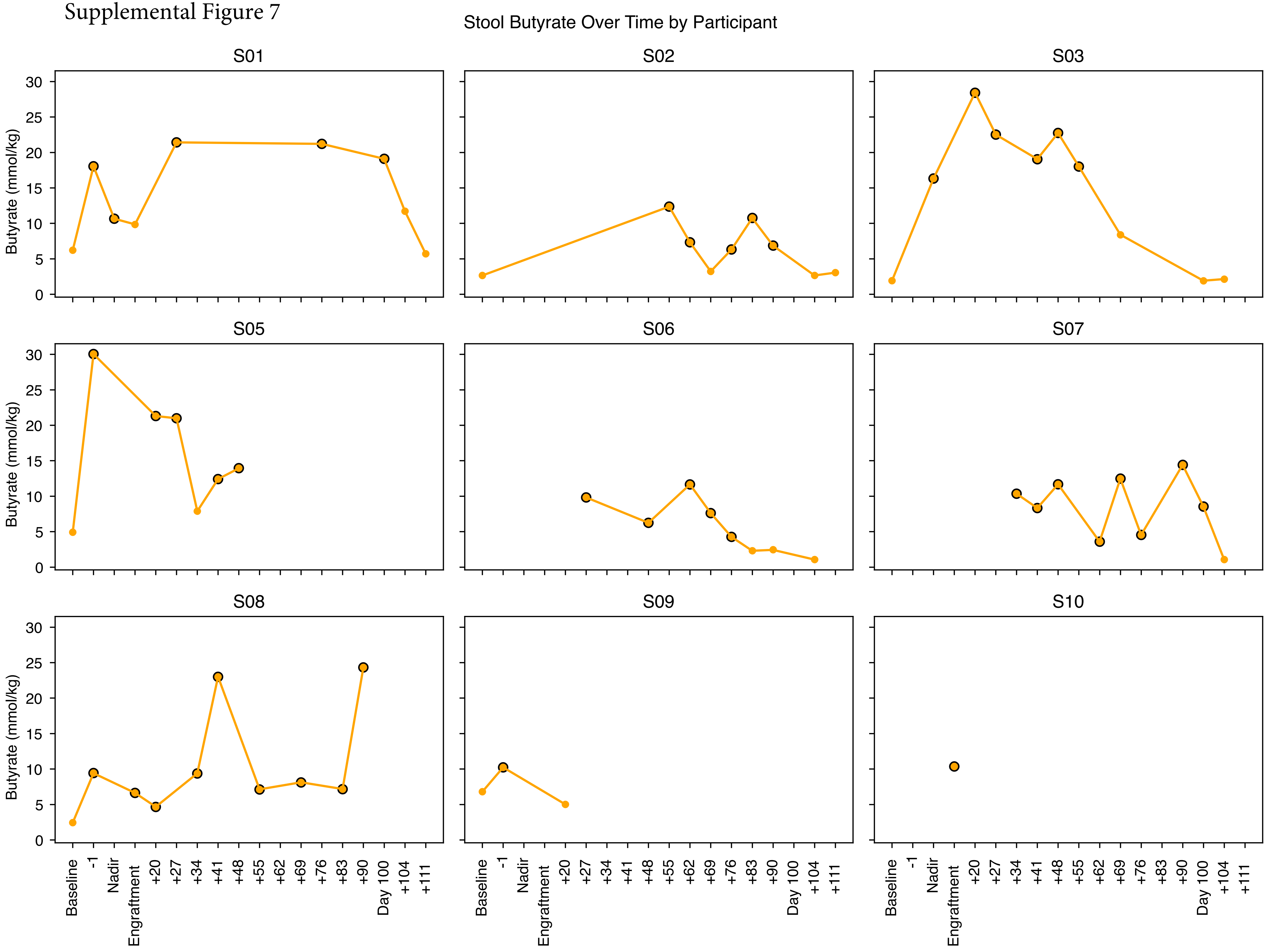

Supplemental Figure legends

Supplemental Figure 1: Stool propionate and acetate over time in allo-HCT recipients

a) To the left, intention to treat analysis of stool propionate levels in mmol/kg (y axis) as measured through allo-HCT where time (x axis) is days relative to allo-HCT. Yellow dots with a black outline represent propionate levels at time points when participants were consuming RPS and yellow dots with no outline represent propionate levels at time points when participants were not taking RPS. Error bars are the intention to treat mean and 95% confidence interval at that time point. To the right, per protocol analysis of stool propionate levels in mmol/kg (y axis) when participants are on RPS versus not (x axis). Mixed random effect model was used to adjust for repeated measures from the same individuals (n=10). Whisker plot represents the mean and the 95% confidence interval of values. b) To the left, intention to treat analysis of stool acetate levels in mmol/kg (y axis) as measured through allo-HCT where time (x axis) is days relative to allo-HCT. Yellow dots with a black outline represent acetate levels at time points when participants were consuming RPS and yellow dots with no outline represent acetate levels at time points when participants were not taking RPS. Error bars are the intention to treat mean and 95% confidence interval at that time point. To the right, per protocol analysis of stool acetate levels in mmol/kg (y axis) when participants are on RPS versus not (x axis). Mixed random effect model was used to adjust for repeated measures from the same individuals (n=10). Whisker plot represents the mean and the 95% confidence interval of values.

Supplemental Figure 2: Microbial compositional changes. a) Change in relative abundance of microbes capable of producing butyrate through allo-HCT comparing historic controls (n=15) to RPS recipients (n=10). Whiskers indicate mean and 95% confidence interval of values based on the SEM. b) Change in relative abundance of RS degraders through allo-HCT comparing historic controls (n=15) to RPS recipients (n=10). Whiskers indicate mean and 95% confidence interval of values based on the SEM.

Supplemental Figure 3: Plasma SCFA levels over time through allo-HCT. Left column are historic control (n=15; blue) and resistant starch (RPS) consuming (n=10; orange) allo-HCT recipients. Whiskers are mean and 95% confidence intervals based on SEM. Center and right columns are the plasma metabolite levels over time per participant (grey), with mean +/- SEM depicted across time. Center is for historic controls (n=15) and right is for RPS recipients (n=10). a) Plasma butyrate levels over time through allo-HCT in historic controls and RPS recipients. b) Plasma propionate levels over time through allo-HCT in historic controls and RPS recipients. c) Plasma acetate levels over time through allo-HCT in historic controls and RPS recipients.

Supplemental Figure 4: Plasma versus stool acetate and propionate. Correlation graphs of plasma and stool acetate and propionate in RPS recipients (n=10) throughout allo-HCT. Only displaying timepoints at which observations of both stool and plasma metabolites were available. A Pearson's correlation was completed for each time point, with a p-value determined based on a two-sided alternative hypothesis given the possibility for negative correlation and against the exact distribution of r.

Supplemental Figure 5: Plasma metabolites over time through allo-HCT. Volcano plots based on student t-test showing changes in plasma metabolites at post allo-HCT timepoints compared to baseline within each of the patients. For all plots, the y-axis is the negative logarithm of the p-value and the x-axis is the logarithm of the fold change between the two timepoints being compared. The top three plots are in the historical control cohort: to the left is a volcano plot of changes in metabolites at nadir compared to baseline, in the middle are changes in metabolites at engraftment compared to baseline, and to the right are changes in metabolites at day 100 compared to baseline. The bottom three plots are in the starch cohort: to the left is a volcano plot of changes in metabolites at nadir compared to baseline, in the middle are changes in metabolites at engraftment compared to baseline, and to the right are changes

in metabolites at day 100 compared to baseline. These plots show changes in plasma metabolites at nadir and engraftment post allo-HCT when compared to baseline independent of whether allo-HCT recipients received RPS.

Supplemental figure 6: RPS-degrader bacteria. Changes in the relative abundance of two populations of RPS-degrader bacteria in allo-HCT patients consuming RPS.

Supplemental figure 7: Stool butyrate over time in allo-HCT by participant. Spaghetti plots for each participant of stool butyrate levels in mmol/kg (y axis) as measured through allo-HCT where time (x axis) in days relative to allo-HCT. Yellow dots with a black outline represent butyrate levels at time points when participants were consuming RPS and yellow dots with no outline represent butyrate levels at time points when participants were not taking RPS.

Supplemental Appendix A:

Clustering and Ordination-based analysis of human plasma metabolomics data

<u>Rationale:</u>
As a complement to association analysis with individual plasma metabolites to outcomes, we also employed an ordination-and-clustering based analysis approach. Our initial motivation was based on [illegible]

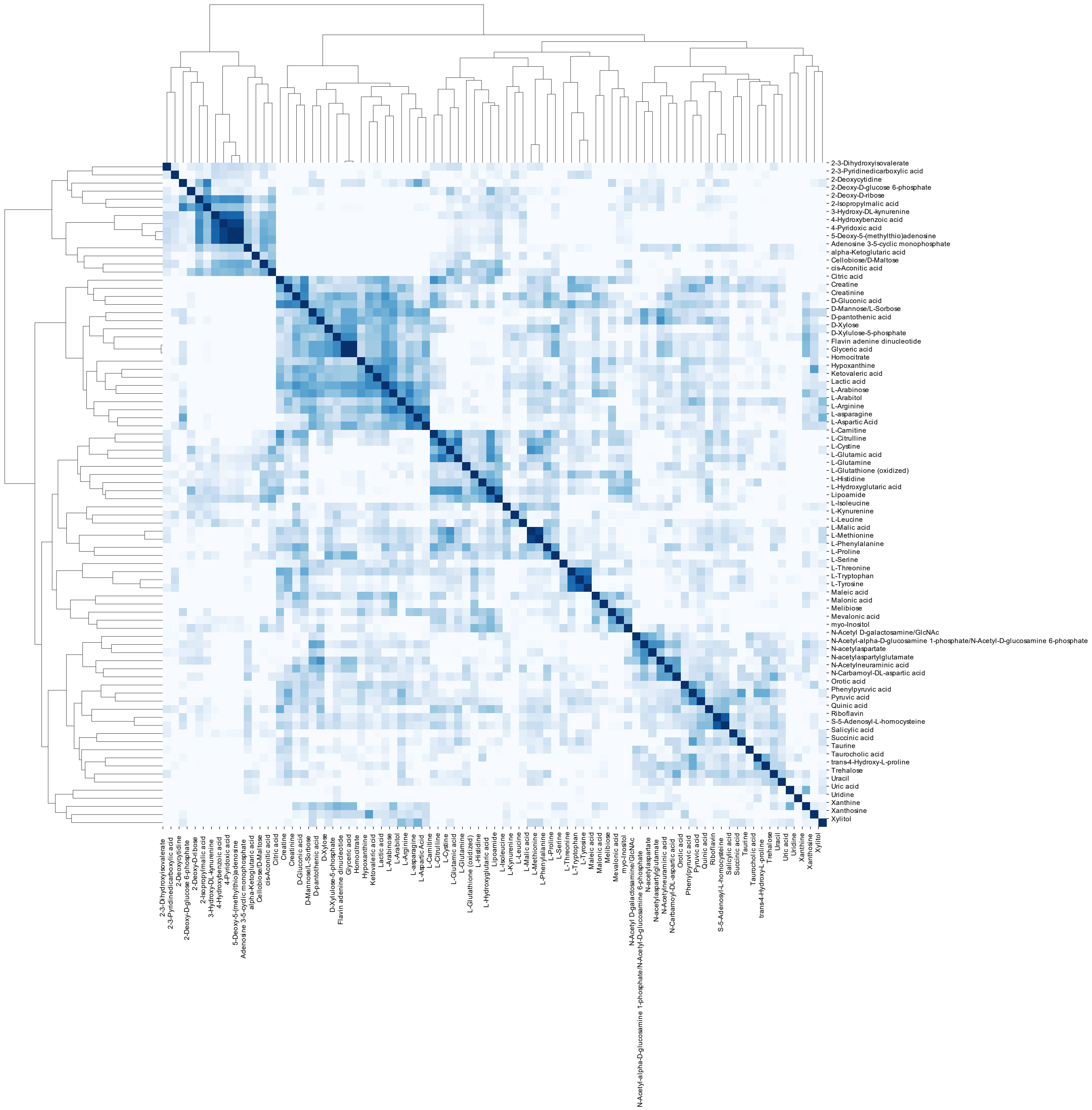

Supplemental Figure 8: Pearson correlation coefficients of median centered plasma metabolite levels reordered by similarity reveal non-independence of multiple observable human plasma metabolites.

The observation that many plasma metabolite levels are highly correlated (**Figure A1**) with one another is perhaps not surprising (i.e., it would be expected that multiple metabolites could be affected by a dysfunctional or missing catabolizing process) and complicates association analysis that often has an assumption of independence. Thus, we hypothesized that plasma metabolite levels may fall into identifiable discrete clusters.

Method:

1. Each plasma metabolites abundance was median centered by dividing the observed abundance by the median observed abundance for that metabolite.

2. Pairwise distance is then calculated between *specimens* using the median-centered plasma metabolite levels. For this study we chose cosine distance as our metric, as it is relatively insensitive to differences in magnitude. Similar results were observed in pilot studies when using Euclidean distances.

3. The pairwise distance matrix is used to ordinate the specimens. For this approach we chose Uniform Manifold Approximation and Projection for Dimension Reduction (UMAP) as the approach[1], in part based on successful employment of this ordination technique in the realm of single-cell data analysis. We made use of the python-umap library, and hyperparameters of 10 nearest-neighbors, two components as output, and a minimum distance of zero.

4. Identification and naming of clusters of plasma metabolites. For this approach we used hdbscan on the embedded points. We used the python hdbscan library, with hyperparameters of min_samples=1 and min_cluster_size=5.

Once each specimen was assigned a cluster, we then:

1. Determined the mean median-centered level of each metabolite by cluster, and then log2 transformed those values.
2. Identified cluster-unique metabolites via GLM: metabolite level ~ cluster, with clusters as dummy variables.
3. Quantify the number of specimens in each cluster within each cohort (historic or starch pilot) and timepoint (Baseline, Nadir, Engraftment, or Day 100) followed by chisquared analysis to establish if the distribution of specimens to clusters at a timepoint are different when comparing between clusters.

Notably, both the ordination-and-clustering and per-metabolite association analysis revealed a similar topline finding: The RPS cohort had more plasma metabolites in a similar state to that observed at baseline at the engraftment timepoint as compared to the historic controls.

Supplementary Appendix B: Butyrate taxons

taxon_id	taxon_name	lineage	rank
2374	Acetonema longum	cellular organisms; Bacteria; Terrabacteria group; Firmicutes; Negativicutes; Selenomonadales; Sporomusaceae; Acetonema	species
905	Acidaminococcus fermentans	cellular organisms; Bacteria; Terrabacteria group; Firmicutes; Negativicutes; Acidaminococcales; Acidaminococcaceae; Acidaminococcus	species
28117	Alistipes putredinis	cellular organisms; Bacteria; FCB group; Bacteroidetes/Chlorobi group; Bacteroidetes; Bacteroidia; Bacteroidales; Rikenellaceae; Alistipes	species
33029	Anaerococcus hydrogenalis	cellular organisms; Bacteria; Terrabacteria group; Firmicutes; Tissierellia; Tissierellales; Peptoniphilaceae; Anaerococcus	species
33032	Anaerococcus lactolyticus	cellular organisms; Bacteria; Terrabacteria group; Firmicutes; Tissierellia; Tissierellales; Peptoniphilaceae; Anaerococcus	species
33034	Anaerococcus prevotii	cellular organisms; Bacteria; Terrabacteria group; Firmicutes; Tissierellia; Tissierellales; Peptoniphilaceae; Anaerococcus	species
33036	Anaerococcus tetradius	cellular organisms; Bacteria; Terrabacteria group; Firmicutes; Tissierellia; Tissierellales; Peptoniphilaceae; Anaerococcus	species
33037	Anaerococcus vaginalis	cellular organisms; Bacteria; Terrabacteria group; Firmicutes; Tissierellia; Tissierellales; Peptoniphilaceae; Anaerococcus	species
214853	Anaerofustis stercorihominis	cellular organisms; Bacteria; Terrabacteria group; Firmicutes; Clostridia; Clostridiales; Eubacteriaceae; Anaerofustis	species
105841	Anaerostipes caccae	cellular organisms; Bacteria; Terrabacteria group; Firmicutes; Clostridia; Clostridiales; Lachnospiraceae; Anaerostipes	species
169435	Anaerotruncus colihominis	cellular organisms; Bacteria; Terrabacteria group; Firmicutes; Clostridia; Clostridiales; Ruminococcaceae; Anaerotruncus	species
84378	Brachyspira murdochii	cellular organisms; Bacteria; Spirochaetes; Spirochaetia; Brachyspirales; Brachyspiraceae; Brachyspira	species
52584	Brachyspira pilosicoli	cellular organisms; Bacteria; Spirochaetes; Spirochaetia; Brachyspirales; Brachyspiraceae; Brachyspira	species
45851	Butyrivibrio crossotus	cellular organisms; Bacteria; Terrabacteria group; Firmicutes; Clostridia; Clostridiales; Lachnospiraceae; Butyrivibrio	species
831	Butyrivibrio fibrisolvens	cellular organisms; Bacteria; Terrabacteria group; Firmicutes; Clostridia; Clostridiales; Lachnospiraceae; Butyrivibrio	species
43305	Butyrivibrio proteoclasticus	cellular organisms; Bacteria; Terrabacteria group; Firmicutes; Clostridia; Clostridiales; Lachnospiraceae; Butyrivibrio	species
1491	Clostridium botulinum	cellular organisms; Bacteria; Terrabacteria group; Firmicutes; Clostridia; Clostridiales; Clostridiaceae; Clostridium	species
1492	Clostridium butyricum	cellular organisms; Bacteria; Terrabacteria group; Firmicutes; Clostridia; Clostridiales; Clostridiaceae; Clostridium	species
1502	Clostridium perfringens	cellular organisms; Bacteria; Terrabacteria group; Firmicutes; Clostridia; Clostridiales; Clostridiaceae; Clostridium	species
84030	[Clostridium] saccharolyticum	cellular organisms; Bacteria; Terrabacteria group; Firmicutes; Clostridia; Clostridiales; Lachnospiraceae; Lachnoclostridium	species
1509	Clostridium sporogenes	cellular organisms; Bacteria; Terrabacteria group; Firmicutes; Clostridia; Clostridiales; Clostridiaceae; Clostridium	species
1512	[Clostridium] symbiosum	cellular organisms; Bacteria; Terrabacteria group; Firmicutes; Clostridia; Clostridiales; Lachnospiraceae; Lachnoclostridium	species
116085	Coprococcus catus	cellular organisms; Bacteria; Terrabacteria group; Firmicutes; Clostridia; Clostridiales; Lachnospiraceae; Coprococcus	species
410072	Coprococcus comes	cellular organisms; Bacteria; Terrabacteria group; Firmicutes; Clostridia; Clostridiales; Lachnospiraceae; Coprococcus	species
33043	Coprococcus eutactus	cellular organisms; Bacteria; Terrabacteria group; Firmicutes; Clostridia; Clostridiales; Lachnospiraceae; Coprococcus	species
29322	[Eubacterium] cellulosolvens	cellular organisms; Bacteria; Terrabacteria group; Firmicutes; Clostridia; Clostridiales; Eubacteriaceae; Eubacterium	species
39484	Agathobaculum desmolans	cellular organisms; Bacteria; Terrabacteria group; Firmicutes; Clostridia; Clostridiales; Ruminococcaceae; Agathobaculum	species
31971	Absiella dolichum	cellular organisms; Bacteria; Terrabacteria group; Firmicutes; Erysipelotrichia; Erysipelotrichales; Erysipelotrichaceae; Absiella	species
39488	[Eubacterium] hallii	cellular organisms; Bacteria; Terrabacteria group; Firmicutes; Clostridia; Clostridiales; Eubacteriaceae; Eubacterium	species
1736	Eubacterium limosum	cellular organisms; Bacteria; Terrabacteria group; Firmicutes; Clostridia; Clostridiales; Eubacteriaceae; Eubacterium	species
39491	[Eubacterium] rectale	cellular organisms; Bacteria; Terrabacteria group; Firmicutes; Clostridia; Clostridiales; Lachnospiraceae; unclassified Lachnospiraceae	species
51123	[Eubacterium] saphenum	cellular organisms; Bacteria; Terrabacteria group; Firmicutes; Clostridia; Clostridiales; Clostridiales incertae sedis; Clostridiales Family XIII. Incertae Sedis	species
39496	Eubacterium ventriosum	cellular organisms; Bacteria; Terrabacteria group; Firmicutes; Clostridia; Clostridiales; Eubacteriaceae; Eubacterium	species
39498	[Eubacterium] yurii	cellular organisms; Bacteria; Terrabacteria group; Firmicutes; Clostridia; Clostridiales; Peptostreptococcaceae; Peptoanaerobacter	species
853	Faecalibacterium prausnitzii	cellular organisms; Bacteria; Terrabacteria group; Firmicutes; Clostridia; Clostridiales; Ruminococcaceae; Faecalibacterium	species
849	Fusobacterium gonidiaformans	cellular organisms; Bacteria; Fusobacteria; Fusobacteriia; Fusobacteriales; Fusobacteriaceae; Fusobacterium	species
850	Fusobacterium mortiferum	cellular organisms; Bacteria; Fusobacteria; Fusobacteriia; Fusobacteriales; Fusobacteriaceae; Fusobacterium	species
851	Fusobacterium nucleatum	cellular organisms; Bacteria; Fusobacteria; Fusobacteriia; Fusobacteriales; Fusobacteriaceae; Fusobacterium	species
861	Fusobacterium ulcerans	cellular organisms; Bacteria; Fusobacteria; Fusobacteriia; Fusobacteriales; Fusobacteriaceae; Fusobacterium	species
856	Fusobacterium varium	cellular organisms; Bacteria; Fusobacteria; Fusobacteriia; Fusobacteriales; Fusobacteriaceae; Fusobacterium	species
1735	Holdemanella biformis	cellular organisms; Bacteria; Terrabacteria group; Firmicutes; Erysipelotrichia; Erysipelotrichales; Erysipelotrichaceae; Holdemanella	species
467210	Lachnoanaerobaculum saburreum	cellular organisms; Bacteria; Terrabacteria group; Firmicutes; Clostridia; Clostridiales; Lachnospiraceae; Lachnoanaerobaculum	species
187326	Megasphaera micronuciformis	cellular organisms; Bacteria; Terrabacteria group; Firmicutes; Negativicutes; Veillonellales; Veillonellaceae; Megasphaera	species
28118	Odoribacter splanchnicus	cellular organisms; Bacteria; FCB group; Bacteroidetes/Chlorobi group; Bacteroidetes; Bacteroidia; Bacteroidales; Odoribacteraceae; Odoribacter	species

1496	Clostridioides difficile	cellular organisms; Bacteria; Terrabacteria group; Firmicutes; Clostridia; Clostridiales; Peptostreptococcaceae; Clostridioides	species
507750	Peptoniphilus duerdenii	cellular organisms; Bacteria; Terrabacteria group; Firmicutes; Tissierellia; Tissierellales; Peptoniphilaceae; Peptoniphilus	species
54005	Peptoniphilus harei	cellular organisms; Bacteria; Terrabacteria group; Firmicutes; Tissierellia; Tissierellales; Peptoniphilaceae; Peptoniphilus	species
33031	Peptoniphilus lacrimalis	cellular organisms; Bacteria; Terrabacteria group; Firmicutes; Tissierellia; Tissierellales; Peptoniphilaceae; Peptoniphilus	species
28123	Porphyromonas asaccharolytica	cellular organisms; Bacteria; FCB group; Bacteroidetes/Chlorobi group; Bacteroidetes; Bacteroidia; Bacteroidales; Porphyromonadaceae; Porphyromonas	species
28124	Porphyromonas endodontalis	cellular organisms; Bacteria; FCB group; Bacteroidetes/Chlorobi group; Bacteroidetes; Bacteroidia; Bacteroidales; Porphyromonadaceae; Porphyromonas	species
837	Porphyromonas gingivalis	cellular organisms; Bacteria; FCB group; Bacteroidetes/Chlorobi group; Bacteroidetes; Bacteroidia; Bacteroidales; Porphyromonadaceae; Porphyromonas	species
281920	Porphyromonas uenonis	cellular organisms; Bacteria; FCB group; Bacteroidetes/Chlorobi group; Bacteroidetes; Bacteroidia; Bacteroidales; Porphyromonadaceae; Porphyromonas	species
556499	Propionibacterium acidifaciens	cellular organisms; Bacteria; Terrabacteria group; Actinobacteria; Actinobacteria; Propionibacteriales; Propionibacteriaceae; Propionibacterium	species
113287	Pseudoramibacter alactolyticus	cellular organisms; Bacteria; Terrabacteria group; Firmicutes; Clostridia; Clostridiales; Eubacteriaceae; Pseudoramibacter	species
301301	Roseburia hominis	cellular organisms; Bacteria; Terrabacteria group; Firmicutes; Clostridia; Clostridiales; Lachnospiraceae; Roseburia	species
166486	Roseburia intestinalis	cellular organisms; Bacteria; Terrabacteria group; Firmicutes; Clostridia; Clostridiales; Lachnospiraceae; Roseburia	species
360807	Roseburia inulinivorans	cellular organisms; Bacteria; Terrabacteria group; Firmicutes; Clostridia; Clostridiales; Lachnospiraceae; Roseburia	species
177972	Shuttleworthia satelles	cellular organisms; Bacteria; Terrabacteria group; Firmicutes; Clostridia; Clostridiales; Lachnospiraceae; Shuttleworthia	species
214851	Subdoligranulum variabile	cellular organisms; Bacteria; Terrabacteria group; Firmicutes; Clostridia; Clostridiales; Ruminococcaceae; Subdoligranulum	species
162	Treponema phagedenis	cellular organisms; Bacteria; Spirochaetes; Spirochaetia; Spirochaetales; Spirochaetaceae; Treponema	species
69710	Treponema vincentii	cellular organisms; Bacteria; Spirochaetes; Spirochaetia; Spirochaetales; Spirochaetaceae; Treponema	species

Supplementary Appendix C: List of Metabolites in Global Snapshot Metabolomics

Detectable metabolites
2-3-Dihydroxyisovalerate
2-3-Pyridinedicarboxylic acid
2-Deoxycytidine
2-Deoxy-D-glucose 6-phosphate
2-Deoxy-D-ribose
2-Isopropylmalic acid
3-Hydroxy-DL-kynurenine
4-Hydroxybenzoic acid
4-Pyridoxic acid
5-Deoxy-5-(methylthio)adenosine
Adenosine 3-5-cyclic monophosphate
alpha-Ketoglutaric acid
Cellobiose/D-Maltose
cis-Aconitic acid
Citric acid
Creatine
Creatinine
D-Gluconic acid
D-Mannose/L-Sorbose
D-pantothenic acid
D-Xylose
D-Xylulose-5-phosphate
Flavin adenine dinucleotide
Glyceric acid
Homocitrate
Hypoxanthine
Ketovaleric acid
Lactic acid
L-Arabinose
L-Arabitol
L-Arginine
L-asparagine
L-Aspartic Acid
L-Carnitine
L-Citrulline
L-Cystine
L-Glutamic acid
L-Glutamine
L-Glutathione (oxidized)
L-Histidine
L-Hydroxyglutaric acid
Lipoamide
L-Isoleucine
L-Kynurenine
L-Leucine
L-Malic acid
L-Methionine
L-Phenylalanine

L-Proline
L-Serine
L-Threonine
L-Tryptophan
L-Tyrosine
Maleic acid
Malonic acid
Melibiose
Mevalonic acid
myo-Inositol
N-Acetyl D-galactosamine/GlcNAc
N-Acetyl-alpha-D-glucosamine 1-phosphate/N-Acetyl-D-glucosamine 6-phosphate
N-acetylaspartate
N-acetylaspartylglutamate
N-Acetylneuraminic acid
N-Carbamoyl-DL-aspartic acid
Orotic acid
Phenylpyruvic acid
Pyruvic acid
Quinic acid
Riboflavin
S-5-Adenosyl-L-homocysteine
Salicylic acid
Succinic acid
Taurine
Taurocholic acid
trans-4-Hydroxy-L-proline
Trehalose
Uracil
Uric acid
Uridine
Xanthine
Xanthosine
Xylitol

UMCC 2016.029
Dietary manipulation of the microbiome-metabolomic axis for mitigating GVHD in allo HCT patients

Principal Investigators:

Mary Mansour Riwes, DO
University of Michigan 1500
E Medical Ctr Dr, MPLAN -
7W, 8A, 8E, Medical School
Ann Arbor MI, 48109-5932
Phone: 734-936-8785
Fax: 734-647-9647
Email: mmriwes@med.umich.edu

Sub-Investigators:

Pavan Reddy, MD
Internal Medicine/Hematology & Oncology

John Magenau, MD
Internal Medicine/Hematology & Oncology

Attaphol Pawarode, MD
Internal Medicine/Hematology & Oncology
Sung Won Choi, MD, MS
Pediatrics/Hematology & Oncology

Sarah Anand, MD
Internal Medicine/Hematology & Oncology

Monalisa Ghosh, MD
Internal Medicine/Hematology & Oncology

Darren King, MD
Internal Medicine/Hematology & Oncology

John Maciejewski, MD
Internal Medicine/Hematology & Oncology

Biostatistician: Thomas Braun, PhD
University of Michigan
1415 Washington Heights, M4063 SPH II
Ann Arbor, MI, 48109-2029
Phone: 734-936-9844
Fax: 734-763-2215
tombraun@umich.edu

Study Intervention: Potato-based dietary starch; Bob's Red Mill® (IND 132208)

TABLE OF CONTENTS

ABBREVIATIONS 5

STUDY SYNOPSIS 6

1.0 BACKGROUND AND RATIONALE 7

1.1 Disease Background 7

1.2 Study Agent Background and Associated Known Toxicities 11

1.3 Rationale 12

2.0 STUDY OBJECTIVES 14

2.1 Primary Objectives 14

2.2 Exploratory Secondary Objectives 14

2.3 Correlative Studies… 14

3.0 PATIENT ELIGIBILITY 15

3.1 Inclusion Criteria 15

3.2 Exclusion Criteria 16

4.0 SUBJECT SCREENING AND REGISTRATION PROCEDURES 16

5.0 TREATMENT PLAN 17

5.1 Treatment Dosage and Administration 17

5.2 Toxicities and Dosing Delays/Dose Modifications 18

5.3 Concomitant Medications/Treatments 18

5.4 Duration of Therapy 18

5.5 Off Treatment Criteria 19

5.6 Duration of Follow-Up 19

5.7 Off Study Criteria 20

5.8 Patient Replacement 20

6.0 STUDY PROCEDURES **21**
6.1 Screening/Baseline Procedures 21
6.2 Procedures During Treatment 22
6.3 Follow-Up Procedures 23
6.4 STUDY CALENDAR 24
7.0 ADVERSE EVENTS **26**
7.1 Experimental Therapy 26
7.2 Adverse Event and Reporting Definitions 26
7.4 Serious Adverse Event Reporting Guidelines 29
7.5 Adverse Event Form Reporting Guidelines 30
7.6 Reporting of Unanticipated Problems 30
8.0 DRUG INFORMATION **31**
9.0 CORRELATIVES/SPECIAL STUDIES **32**
9.1 Sample Collection Guidelines 33
9.2 Specimen Banking 33
10.0 STATISTICAL CONSIDERATIONS **34**
10.1 Study Design 34
10.2 Sample Size and Accrual 34
10.3 Data Analyses Plans 35
11.0 DATA AND SAFETY MONITORING **35**
12.0 REFERENCES **36**
13.0 APPENDICES **38**

ABBREVIATIONS

AE	Adverse Event
ALT	Alanine Aminotransferase
ALC	Absolute Lymphocyte Count
AST	Aspartate Aminotransferase
BUN	Blood Urea Nitrogen
CBC	Complete Blood Count
CMP	Comprehensive Metabolic Panel
CTCAE	Common Terminology Criteria for Adverse Events
CTO	Clinical Trials Office
DSMB	Data and Safety Monitoring Board
GVHD	Graft versus Host Disease
H&P	History and Physical
HLA	Human Leukocyte Antigen
HRPP	Human Research Protections Program
IEC	Intestinal epithelial cell
IND	Investigational New Drug
IRB	Institutional Review Board
IV (or iv)	Intravenously
NCI	National Cancer Institute
PBMCs	Peripheral Blood Mononuclear Cells
PI	Principal Investigator
p.o.	per os/by mouth/orally
PRC	Protocol Review Committee
SAE	Serious Adverse Event
SCFA	Short Chain Fatty Acid
SCT	Stem Cell Transplant
SGOT	Serum Glutamic Oxaloacetic Transaminase
SPGT	Serum Glutamic Pyruvic Transaminase
TRM	Transplant Related Mortality
UaP	Unanticipated Problem
WBC	White Blood Cells

STUDY SYNOPSIS

Title	Modification of the Intestinal Microbiome by Diet Intervention to Mitigate Acute Graft-Versus-Host Disease
Phase	Phase II
Methodology	Pilot study testing feasibility of dietary intervention followed by randomized, blinded controlled trial
Study Duration	Nine years
Study Center(s)	Single-center
Objectives	1. Assess feasibility and tolerability of a starch-based dietary supplement in patients undergoing allogeneic SCT.<br>2. Assess impact of diet on incidence of acute GVHD and infectious complications.<br>3. Gain an understanding of the dynamic changes of the intestinal microbiome and metabolome in subjects undergoing allogeneic SCT.
Number of Subjects	70 evaluable subjects (10 for feasibility pilot; 60 for phase II component randomized in a 5:1 fashion)
Inclusion Criteria	Patients undergoing matched related full intensity allogeneic stem cell transplant
Exclusion Criteria	IBD, history of gastric bypass surgery, active Clostridium difficile infection, active participation in alternative GVHD prevention trial, any physical or psychological condition that, in the opinion of the investigator, would post unacceptable risk to the patient or raise concern that the patient would not comply with protocol procedures
Study Product(s), Dose, Route, Regimen	Potato-based dietary starch, Bob's Red Mill® (IND 132208)<br>20 g orally twice daily
Duration of Administration	108 days
Reference Therapy	Standard SCT diet
Statistical Methodology	This is a two-phase study. The first phase will assess feasibility of administration of this dietary supplement to 10 subjects undergoing allogeneic SCT and its effect on the structure of recipients' intestinal microbiome and its metabolites, particularly the short chain fatty acid (SCFA), butyrate. Feasibility is defined as ability to take 70% or more of scheduled doses in 60% or more patients. The second phase of the study will assess efficacy of this dietary supplement in preventing acute GVHD in 60 subjects. The primary endpoint will be the estimated cumulative incidence of acute GVHD. This portion will contain 50 subjects receiving the dietary intervention and 10 randomized controls. This sample size was chosen to produce a 95% confidence interval with a half-width of no more than 14 points.

1.0 BACKGROUND AND RATIONALE

1.1 Disease Background

Allogeneic Stem Cell Transplant Background:

Allogeneic stem cell transplantation (SCT) is an intensive treatment modality that often represents the only curative therapy for patients with aggressive hematologic malignancies or other marrow failure syndromes. More than 8,000 allogeneic stem cell transplants were performed in the United States in 2013 according to CIBMTR data (Pasquini MC 2014). The number of allogeneic SCTs performed is increasing owing to improvements in donor selection, conditioning regimens and supportive care. Despite this, the procedure continues to carry a high morbidity and mortality. One of the principle contributors to transplant related mortality (TRM) is graft versus host disease (GVHD).

Graft Versus Host Disease:

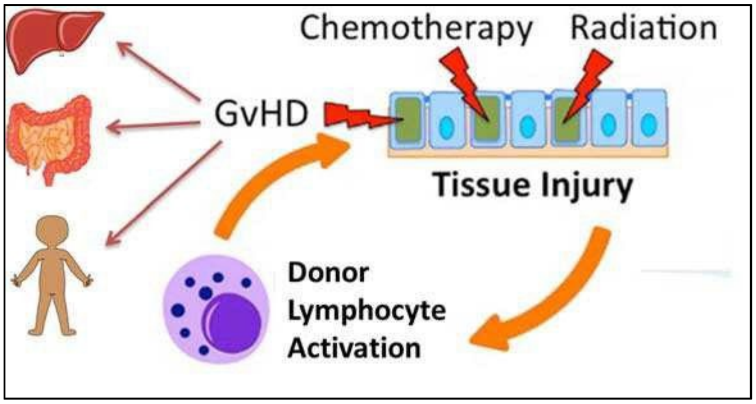

Figure 1: Pathophysiology of GVHD. (Brennan*, et al* 2012)

GVHD develops in approximately 40-50% of patients undergoing HLA matched related SCT and 50-70% of recipients receiving unrelated donor SCT (Ferrara*, et al* 2009, Lee*, et al* 2007, Nash*, et al* 1996, Ratanatharathorn*, et al* 1998) and proves fatal to 15% of transplant recipients (Chen*, et al* 2015). One of the reasons for this high mortality is that once established, GVHD can be resistant to front-line treatment with corticosteroids in more than 50% of patients (Deeg 2007). Survival is significantly diminished for patients with steroid refractoriness or those in whom treatment is prolonged (Gomez-Almaguer*, et al* 2008, Levine*, et al* 2010, MacMillan*, et al* 2010).

GVHD is an immunological phenomenon whereby donor lymphocytes respond to polymorphic HLAs present on host tissues by mounting an attack against these tissues. This results in a clinical syndrome that can be described as an inflammatory response directed predominantly against host antigens in the skin, intestine and liver (Ferrara*, et al* 2009). The interaction between donor lymphocytes and polymorphic HLAs on these host tissues is amplified by the significant tissue injury that occurs in transplant recipients as a result of the conditioning regimen, see **Figure 1.** As a

result, patients manifest clinically significant diarrhea and mucositis in the days and weeks immediately following allogeneic SCT.

Intestinal Microbiome:

The role of the intestinal microbiome is increasingly being examined in a variety of inflammatory conditions. There is research linking changes in the microbiome to conditions as varied as obesity, atherosclerosis, chronic kidney disease, and inflammatory bowel disease (Goldsmith and Sartor 2014). Given the partial overlap in the immune biology of acute GVHD and other inflammatory conditions, the role of the intestinal microbiome in allogeneic SCT is now being investigated.

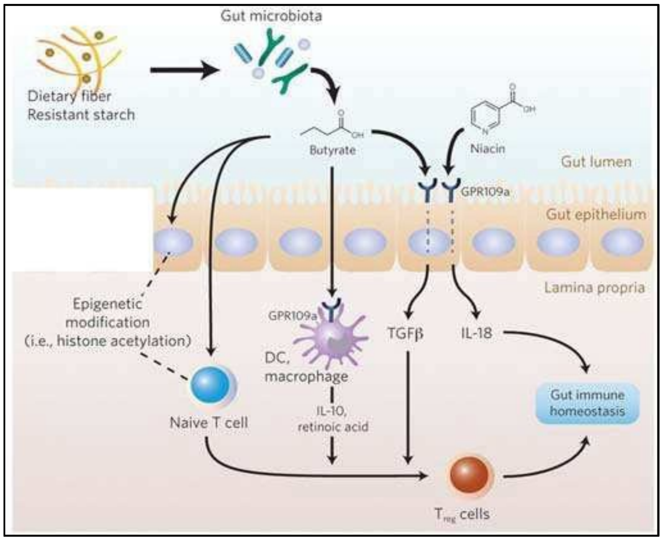

Figure 2. Metabolism of resistant starch by intestinal microbiome and role in intestinal homeostasis

Biological Rational:

Experimental Data:

The process of allogeneic SCT has been shown to result in alteration of the intestinal microbiome (Chen, *et al* 2015). While changes to the composition of the microbiome may have several implications, one is resulting changes in the metabolic milieu of the intestine, with resulting effects on the maintenance of intestinal mucosal homeostasis (Chen, *et al* 2015). Specifically, the intestinal mucosa is comprised of intestinal epithelial cells (IECs) that function to physically segregate commensal bacteria and regulate the intestine's barrier function (Peterson and Artis 2014). In preliminary data from our laboratory, IECs utilize short chain fatty acids (SCFAs)as a primary energy source (Mathewson, *et al* 2016). SCFAs are produced by fermentation of non-digestible carbohydrates by anaerobic bacteria within the colon (Goldsmith and Sartor 2014), **Figure 2.** Butyrate is one prototypical SCFA that has been associated with inflammatory conditions of the bowel (Goldsmith and Sartor 2014).

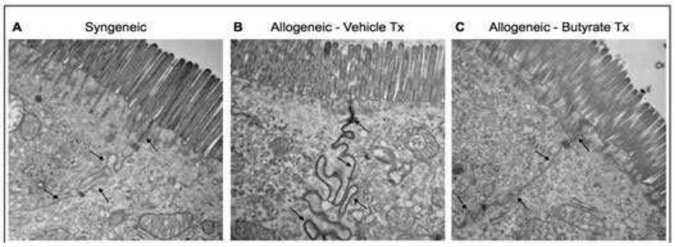

Figure 3. Electron microscopic images of IEC junction taken 7 days post-syngeneic SCT (A), allogeneic SCT (B), and allogeneic SCT treated with butyrate (C).

Previous work in a murine model of GVHD has demonstrated decreased levels of butyrate within IECs after allogeneic HCT. In addition, the IECs from these mice were noted to have decreased receptors for butyrate. Furthermore, exogenous administration of butyrate resulted in increased levels of butyrate within the IECs and improvement in the junctional integrity of intestinal epithelium (Mathewson*, et al* 2016), **Figure 3.** Furthermore, mice receiving allogeneic SCT and supplied with exogenous butyrate lived longer when compared to mice treated with placebo, **Figure 4.** Butyrate has also been demonstrated to function as a histone deacetylase (HDAC) inhibitor (Chang*, et al* 2014). HDAC inhibitors have recently been shown to decrease rates of clinical GVHD via down-regulation of antigen presenting cells and up-regulation of donor T regulatory cells (Choi*, et al* 2014).

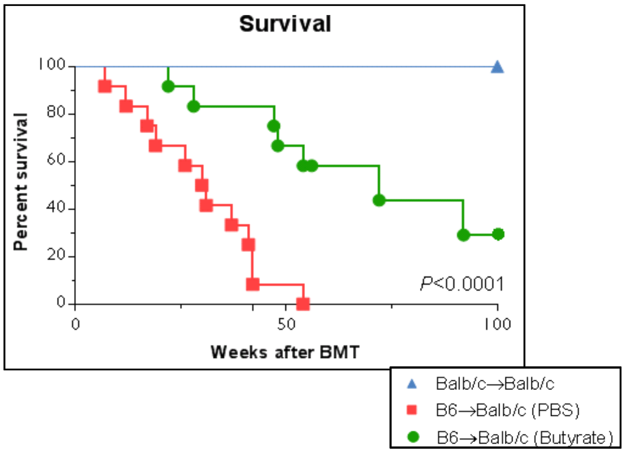

Figure 4. Impact of butyrate treatment on survival of mice undergoing allogeneic transplant.

Preliminary Data from Healthy Humans:

Given this exciting preclinical work, we are interested in studying the intestinal microbiome and intestinal metabolic milieu in patients undergoing allogeneic SCT. We postulate that levels of butyrate are diminished in these patients and are interested in restoring these levels as a means to maintain intestinal mucosal homeostasis. Direct dietary administration of butyrate is impractical given its poor pharmacological properties (e.g. short half-life) and the multigram doses needed to achieve therapeutic concentrations. While increased butyrate levels could be accomplished by trying to directly alter the microbiome with interventions that alter concentrations of butyrate-producing bacteria (Atarashi*, et al* 2013) – a "probiotic approach", an alternative method would be

to simply increase the delivery of non-digestible carbohydrate to the existing microbiome, or a "prebiotic approach." This second approach is particularly appealing when considering the immunocompromised stem cell transplant population, in whom the prospect of directly manipulating the microbiome with administration of even commensal bacteria raises important safety concerns. However, a dietary intervention, through providing the necessary food source, may ultimately provide favorable conditions for beneficial SCFA-producing commensals. As an initial proof of concept, administration of a corn-based resistant starch, to healthy volunteers has shown it to be safe and efficacious in increasing fecal butyrate, **Figure 5**.

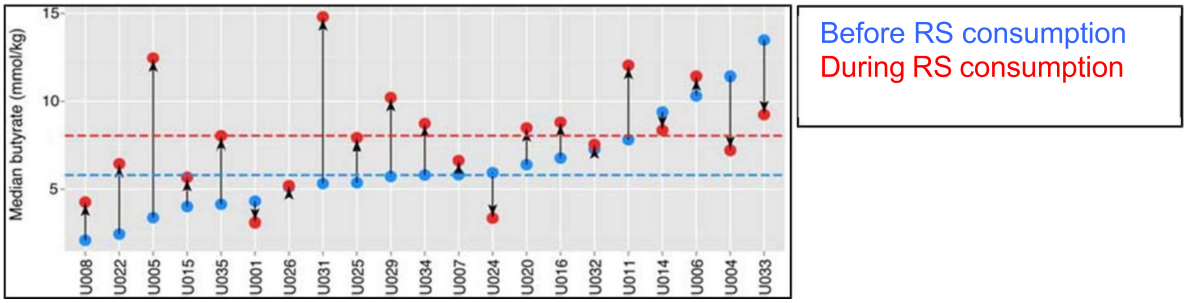

Figure 5. Changes in stool butyrate level pre- and post- consumption of resistant starch.

To better evaluate which of many commercially available resistant starches might be most efficacious in increasing stool butyrate level over a short period of administration, we conducted similar studies using several starch sources, potato-based starch; corn-based starch (HM260); inulin a soluble plant-based starch, and arabinoxylan (ara/xyl) another plant based starch. By doing these additional studies, we were able to demonstrate that the potato-based starch was most effective at increasing fecal butyrate levels, as demonstrated in **Figure 6.** We have therefore elected to proceed with this potato-based starch for further investigation

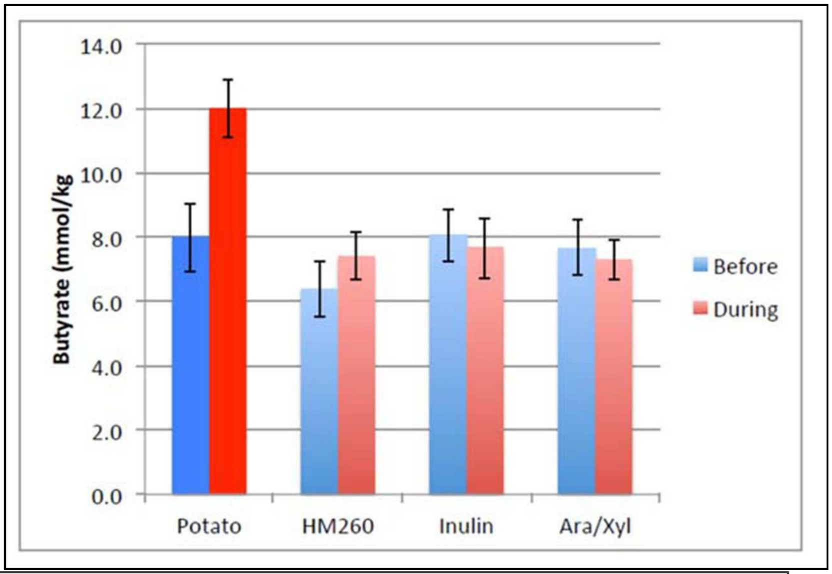

Figure 6. Changes in stool butyrate level after administration of various resistant starches.

Study Goals and Objectives:

We are planning on evaluating the feasibility, safety and early efficacy of administering a commercially available dietary supplement containing potato-based resistant starch to subjects undergoing allogeneic SCT. The intervention will begin immediately prior to the conditioning phase and continue through day 100. Our hypothesis is that a short term administration of a resistant starch is capable of increasing levels of butyrate within the intestine that will reduce rates of acute GVHD. We will achieve these goals through the following objectives:

1. Monitoring the longitudinal changes of the intestinal microbiome before and after administration of a resistant starch in the setting of allogeneic SCT
2. Monitoring the dynamic changes of the metabolic milieu in the setting of allogeneic SCT
3. Assessing the tolerability of a dietary supplement in the allogeneic SCT population
4. Measuring the effect of administration of a resistant starch on the levels of fecal butyrate
5. Assessing the effects of a resistant starch on rates of acute GVHD at day 100

1.2 Study Agent Background and Associated Known Toxicities

The potato-based starch produced by Bob's Red Mill® (IND 132208) is a commercially available low-

digestible carbohydrate. It is entirely plant based and not genetically modified. It is gluten free. It contains approximately 50% slowly-digestible starch by weight, which is digested within the small intestine and slowly absorbed as glucose. The remaining 50% is resistant starch, which is not digested in the small intestine. This resistant starch component reaches the large intestine where it is fermented by bacteria and produces SCFAs. It contains 40 calories per 12 gram serving and no additional dietary nutrients, vitamins or sodium (labelling information).

Potato based starch has been well studied within the dietary and nutritional literature and has been demonstrated safe. (Cummings*, et al* 1996, Ek*, et al* 2014, Kaur*, et al* 2011, Raben*, et al* 1994, Slavin 2013).

In addition, it has previously been administered to healthy human volunteers at the University of Michigan as part of HUM00103995 – Linking the Structure and Function of the Gut Microbiome. This study has revealed no adverse effects in these subjects.

1.3 Rationale

Short chain fatty acids, such as butyrate, have been shown to play an integral role in intestinal epithelial homeostasis. SCFAs are typically metabolized from non-fermentable starches by a healthy intestinal microbiome. As the intestinal microbiome is altered in SCT, we hypothesize that levels of SCFA, namely butyrate, may be decreased. This has previously been demonstrated in an established murine model of GVHD (Mathewson*, et al* 2016).

We postulate that in patients undergoing allogeneic SCT, alterations occur in the intestinal microbiome that may provoke the onset of GVHD.

- These alterations in the microbiome will lead to changes in the intestinal metabolic milieu, specifically decreased levels of SCFAs
- Diminished levels of SCFAs will impair intestinal mucosal homeostasis leading to a less resilient intestinal epithelium
- Which places certain individuals with an increased propensity to develop more extensive tissue damage at heightened risk for developing acute GVHD

Our study aims to study changes in stool microbiome content early after SCT as outlined above as well as to perform a dietary intervention with an exogenous source of resistant starch that facilitates host production of butyrate. As outlined below, we will evaluate whether this is a feasible and tolerable intervention in a medically complex patient population. Second, through frequent monitoring of the intestinal microbiome via stool samples of subjects both pre- and post- transplantation, we will be able to assess the dynamic changes of the intestinal microbiome and metabolome that occur over time in the SCT setting.

Specifically, we will learn what effect this dietary intervention has on stool butyrate levels in comparison to contemporaneous control subjects who do not receive the dietary intervention. Finally, we will assess the incidence of acute GVHD at day 100 post SCT in patients receiving this dietary intervention.

STUDY SCHEMA

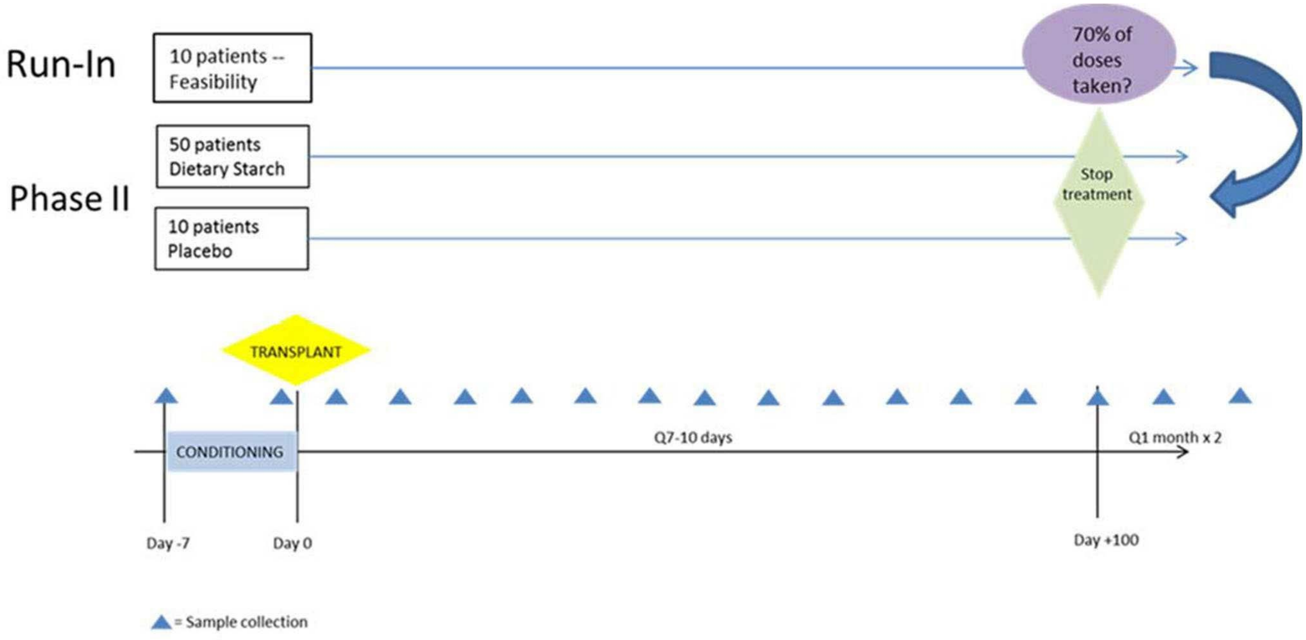

Figure 7: Proposed Study Schema

Initially we will perform a 'run in' phase that will enroll 10 evaluable **adult** subjects undergoing matched relatedfull intensity allogeneic SCT for a hematologic malignancy. While the phase II study will be open to adult **and** pediatric subject, see section 3.1.2 below, the initial feasibility portion will be limited toadult patients only as we do not want to declare this intervention non-feasible based solely on theunique challenges in providing children with a new food stuff. All 10 subjects will receive a potato-starch produced by Bob's Red Mill® (IND 132208). Initially, subjects will take 20 g daily for firstthree days prior to increasing dose to 20 g BID. The reason for providing a smaller dose for theinitial three days is to allow for a "run in" period to allow subjects and their intestinal microbiometime to adapt to this new resistant starch and to improve tolerability and likelihood that subjects willbe able to remain on study. This dosing is supported by the experience of the healthy volunteersin HUM00103995. All subjects will begin taking the potato starch 7 days prior to transplant (day - 7) and continue taking this supplement through 100 days after transplant (day +100). Registereddieticians who work primarily with the SCT population (SB and SP) will work closely with subjectsand study investigators to provide the potato starch in

a way that is most palatable. Study

Coordinator will closely work with patients to document use of the supplement in the electronic medical record post hospitalization and unused supplement will be returned and documented.

Stool samples will be collected serially in the pre- and post-engraftment phases. We will collect stool at time of admission prior to starting conditioning, prior to SCT (day -1), one week post- transplant (day +7), and at time of engraftment (i.e. approximately day +10-day +17). Following engraftment samples will be collected every 7-10 days through day 100. After day 100, two additional stool samples will be collected at monthly intervals. These samples will be used to assess intestinal microbiome and metabolome, including fecal butyrate levels. This portion of the study will serve to establish feasibility as assessed by the tolerability of the dietary supplement for subjects and providers' ability to perform the necessary stool studies in order to assess the effect of the dietary supplement on the structure of recipients' intestinal microbiome and its metabolites, particularly the short chain fatty acid (SCFA), butyrate. Tolerability will be defined as ability to take greater or equal to 70% of scheduleddoses in six or more of the patients enrolled. While inpatient, this will be documented in the electronic medical record by the care team. As an outpatient, teh study coordinator will closely work with patients todocument doses taken in the electronic medical record. Subjects will be monitored for potential side effects such as bloating, gas, or generalized anorexia which may make compliance difficult.

Following this feasibility run-in, if documented safe and tolerable, we will begin the second phase of our study. We will plan on enrolling an additional 50 evaluable subjects to receive the resistant potato- starch on the same schedule noted above and with the same assistance from dieticians and 10 evaluable subjects who receive iso-caloric, non-resistant starch placebo which will serve as contemporaneous controls specifically for studies of the microbiome (5:1 randomization). Again, stool samples will be collected on admission for conditioning, prior to transplant, approximately one week post-transplant, after engraftment and then q7-10days through day 100. After day 100, two additional stool samples will be collected at monthly intervals.

2.0 STUDY OBJECTIVES

2.1 Primary Objectives

To measure the incidence of grade II-IV GVHD as documented on day 100

2.2 Exploratory Objectives

2.2.1 To measure the rates of active Clostridium difficile infection

2.2.2 To evaluate overall survival (OS) and disease free survival (DFS) at 1 year following HCT

2.2.3 To measure fecal and plasma butyrate and levels of other stool

a n d p l a s m a metabolites insubjects undergoing allogeneic SCT.

2.2.4 To describe the tolerability of a dietary supplement in subjects undergoing an allogeneic SCT

2.2.5 To describe the changes in the intestinal microbiome in subjects undergoing allogeneic

SCT

2.3 Correlative Studies

2.3.1 To examine functional responses of antigen presenting cells and T cells from peripheral

blood mononuclear cells (PBSC) before and after administration of potato- starch, produced by Bob's Red Mill® (IND 132208)

2.3.2 To perform phenotyping of T cells, T regulatory cells (Tregs), B cells, NK cells and other cellular immune subsets from peripheral blood mononuclear cells (PBSC) before and after donor cell infusion (HCT)

2.3.3 To assess plasma concentrations of pro-inflammatory cytokines, damage-associated molecular patterns (DAMPs) and GVHD biomarkers before and after administration of potato-starch

2.3.4 In patients undergoing a clinically indicated colonoscopy, obtain an intestinal biopsy tissue for research purposes in order to measure metabolites in IECs and expression of butyrate receptors

3.0 PATIENT ELIGIBILITY

Subjects must meet all of the inclusion and exclusion criteria to be enrolled to the study. Study treatment may not begin until a subject is enrolled.

3.1 Inclusion Criteria

3.1.1 Subjects undergoing matched related full intensity allogeneic HSCT

3.1.2 Age ≥ 18 years for the feasibility phase. Age ≥10 years old AND ≥50 kg for the phase II portion.

3.1.3 Karnofsky >70%, see Appendix A

3.1.4 Subjects must be able to swallow capsules/tablets

3.1.5 Ability to understand and the willingness to sign a written informed consent

3.1.6 Availability of an HLA matched related donor

3.1.7 Willingness to consent / co-enroll on BMT long term follow up study or HUM00043287 (UMCC2001-0234).

3.2 Exclusion Criteria

3.2.1 Patients with inflammatory bowel disease

3.2.2 Patients with a history of gastric bypass surgery

3.2.3 Patients with active Clostridium difficile infection at the time of study enrollment. Active infection is defined as a stool sample positive for Clostridium difficile toxin via EIA and either symptoms (frequent loose stools) OR imaging findings consistent with toxic megacolon

3.2.4 Patients actively enrolled on any other GVHD prevention trial

3.2.5 Any physical or psychological condition that, in the opinion of the investigator, would post unacceptable risk to the patient or raise concern that the patient would not comply with protocol procedures

4.0 SUBJECT SCREENING AND REGISTRATION PROCEDURES

This study will be conducted at the University of Michigan. An IRB-approved informed consent must be

obtained from patients (or legal guardians) prior to the initiation of treatment on this protocol. After informed consent is obtained and PRIOR to the initiation of protocol therapy all patients satisfying the inclusion/exclusion criteria must have eligibility confirmed by the PI or Co-I of the study team.

The patient will not be considered enrolled in the study until all information is confirmed by the PI or Co-I.

5.0 TREATMENT PLAN

5.1 Treatment Dosage and Administration

Protocol treatment must start within 30 business days of enrollment to the study.

5.1.1 Subjects will receive standard BMT diet plus potato- based resistant starch produced by Bob's Red Mill® (IND 132208) or standard BMT diet plus placebo (accessible starch) beginning on day -7 and continuing through day +100. Treatment will be oral and will be administered as both an inpatient (during conditioning, transplant, and recovery phase) as well as an outpatient. Inpatient administration will be documented in the electronic medical record by the care team.. After switching to outpatient administration, the study coordinator will closely work with patient to document use of the supplement in the electronic medical record.. No monitoring of vital signs during or after administration is required. No pre-medications are necessary. There is no emetogenic risk. No known drug interactions. Missed (or vomited) doses will simply be omitted with no need to "make up" a dose.

**Agent**	**Pre-medications; Precautions**	**Emetogenic risk**	**Dose**	**Route**	**Schedule**
Potato-starch	None	None	20 g, (daily for first 3 days followed by BID)	PO	Day -7 through day 100

5.2 Toxicities and Dosing Delays/Dose Modifications

Any patient who receives treatment on this protocol will be evaluable for toxicity. Each patient will be assessed for the development of toxicity according to the Time and Events Table (Section 6.4). Toxicity will be assessed according to the NCI Common Terminology Criteria for Adverse Events (CTCAE), version 4.0. It is anticipated that patients undergoing allogeneic SCT will have significant toxicity that is not attributable to the study agent. Given the investigational agent is a dietary supplement that is a key component of the 2015 FDA Dietary Guidelines (http://health.gov/dietaryguidelines/2015/guidelines/chapter-1/key-recommendations/) and has previously been well-studied in other healthy volunteers; we have no expectation of any hematologic, hepatic or renal toxicity related to this agent. The primary potential toxicity which we will monitor for closely is the development of GI toxicity related to the ingestion of a highly resistant starch. This may be manifested by bloating, flatulence, or GI discomfort that results in intolerance of the dietary supplement. This is because patients in the early post- SCT period may be generally intolerant to the majority of foodstuffs due to ongoing medications, prior chemotherapy and persistent nausea related to the SCT process itself. If such intolerability occurs, a dose reduction can be made to once daily instead of BID dosing at the discretion of the treating provider and/or patient. No other dose modifications will be made beyond this. If intolerability continues, despite decreasing the frequency of dosing, investigational agent may be held for up to 14days. Investigational agent can also be held for up to 14 days if the patient is NPO for anyreason. Subjects who leave the study prior to day 30 due to relapse of primary malignancy and/or toxicities deemed not related to resistant starch supplementation are eligible to be replaced at the discretion of the PI. Subjects who leave more than 30 days into the study, are not eligible to be replaced and will be included with an intention to treat protocol.

5.3 Concomitant Medications/Treatments

No concomitant medications or treatments are prohibited on this study. Specifically there is no prohibition of concomitant antibiotic, antiviral or antifungal therapy. Subjects may co-enroll on other investigational studies except for investigational studies whose primary aim is the prevention of GVHD.

5.4 Duration of Therapy

Therapy will continue for 108 days, i.e. prior to SCT and through day 100 post-SCT, or until one of the following criteria apply:

- Inter-current illness that prevents further administration of treatment. This can

include severe GVHD that renders patient NPO for >14 days.

- Unacceptable adverse event(s) such as severe GI upset or flatulence thought to be related to the dietary supplement.
- Subject suffers a relapse of primary hematologic malignancy.
- Patient voluntarily withdraws from treatment **OR**
- General or specific changes in the patient's condition render the patient unacceptable for further treatment in the judgment of the investigator

5.5 Off Treatment Criteria

Patients will be removed from protocol therapy when any of the criteria listed in Section 5.4 apply. Document in the source the reason for ending protocol therapy and the date the patient was removed from treatment. All patients who discontinue treatment should comply with protocol specific follow-up procedures as outlined in Section 5.6. The only exception to this requirement is when a subject withdraws consent for all study procedures or loses the ability to consent freely.

5.6 Duration of Follow-Up

Subjects will be followed for a total of one year from the date of allogeneic SCT or until death, whichever occurs first. Follow-up will be dictated by the standard operating protocol in the post-allogeneic SCT setting. This will include frequent clinical visits with the primary transplant physician in the first 100 days post allogeneic SCT. Following day 100, patient will be considered off treatment and in the follow-up period until one year. However, collection of two additional stool samples will be required after day + 100; one at approximately day +130 and the second at approximately day +160. A review of the electronic medical record and/or a telephone call will be performed to ascertain information regarding subject status at one-year post SCT.

The follow-up procedures after day 100 including collection of stool specimens outlined above are either standard of care for patients undergoing SCT or part of feasibility assessments for this protocol, Furthermore, they reflect exploratory secondary endpoints and such missing events will be recorded but will not constitute a protocol deviation.

5.7 Off Study Criteria

Patients can be taken off study at any time at their own request, or they may be withdrawn at the discretion of the investigator for safety, behavioral or administrative reasons. The reason(s) for discontinuation from study will be documented and may include:

5.7.1. Patient withdraws consent (termination of treatment and follow-up);

5.7.2. Loss of ability to freely provide consent through imprisonment or involuntary incarceration for treatment;

5.7.3. Patient is unable to comply with protocol requirements;

5.7.4. Treating physician judges continuation on the study would not be in the patients best interest;

5.7.5. Patient becomes pregnant (pregnancy to be reported along same timelines as a serious adverse event);

5.7.6. Development of second malignancy (except for basal cell carcinoma or squamous cell carcinoma of the skin) that requires treatment, which would interfere with this study;

5.7.7. Lost to Follow-up. If a research subject cannot be located to document survival after a period of 1 years, the subject may be considered “lost to follow-up.” All attempts to contact the subject during the one year period must be documented.

5.7.8. Termination of the study by The University of Michigan;

5.7.9. Patient completes protocol treatment and follow-up criteria.

5.8 Patient Replacement

If subject needs to go off study due to one of the criteria delineated in section 5.7, protocol dictates that subject can be replaced if < day 30from transplant.. If >30 transplant days into study protocol, subject data will be included in an intention to treat protocol and will notbe replaced.

6.0 STUDY PROCEDURES

6.1 Screening/Baseline Procedures

Assessments performed exclusively to determine eligibility for this study will be done only after obtaining informed consent. Assessments performed for clinical indications (not exclusively to determine study eligibility) may be used for baseline values even if the studies were done before informed consent was obtained.

All screening procedures must be performed within 30 days prior to registration unless otherwise stated. The screening procedures represent BMT institutional standards of care, which include:

6.1.1 Informed Consent

6.1.2 Medical history

Complete medical and surgical history, history of infections

6.1.3 Demographics

Age, gender, race, ethnicity

6.1.4 Review subject eligibility criteria

6.1.5 Review previous and concomitant medications

6.1.6 Physical exam including vital signs, height and weight

Vital signs (temperature, pulse, respirations, blood pressure), height, weight

6.1.7 Performance status

Performance status evaluated prior to study entry according to Appendix A.

6.1.8 Adverse event assessment

Baseline adverse events will be assessed. See Section 8.0 for Adverse Event monitoring and reporting.

6.1.9 Hematology

6.1.10 Blood draw for correlative studies

See Section 9.0 for details.

6.1.11 Serum chemistries

Comprehensive metabolic panel (CMP) to include: albumin, alkaline phosphatase, ALT/SGPT, AST/SGOT, BUN, creatinine, electrolytes (sodium, potassium, calcium, chloride, bicarbonate), glucose, and total bilirubin.

6.1.12 Pregnancy test (for females of child bearing potential)

6.2 Procedures During Treatment

6.2.1 Stool Samples

- Day -7 (+/- 3 day)
- Day -1 (+/- 3 day)
- Day +7 (+/- 3 days)
- At time of engraftment
- Q7-10 days from time of engraftment through day +100 (+/- 7days)
- Day +100 (+/- 7 days)
- Day +130 (+/- 7 days)

- Day + 160 (+/- 7 days)

6.2.2 Correlative Studies

- Day -7 (+/- 3 day)
- At time of engraftment
- Day +100 (+/- 7 day)

6.2.3 Assessment of GVHD at day 100, +/- 7 days

- Physical exam, vital signs, history
- Hematology
- Serum chemistries

6.3 Follow-Up Procedures

Two additional stool samples will be collected after day + 100; one at approximately day +130 and the second at approximately day +160. A review of the electronic medical record and/or a telephone call will be performed to ascertain information regarding subject status at one year postSCT. Subjects will not require any protocol specific follow-up after one year

6.4 Study Calendar

	Pre-Study	Treatment Period					Follow-Up Period			
**Observations**	Enrollment	Day -7 +/- 3 days	Day -1 +/- 3 days	Day +7 +/-3 days	Engraftment	Q7-10 days through day +100 (+/- 7days)	Day +100 +/- 7 days	Day +130 +/- 7 days	Day +160 +/- 7 days	Day +365 +/- 21 days
Informed Consent	X									
H&P	X									
Pre-HCT Organ Function Testing[1]	X									
KPS	X									
Pregnancy test	X									
Laboratory Testing[2]	X		X		X					
Study Agent		X	X	X	X	X				
Stool Sample		X	X	X	X	X	X	X	X	
Acute GVHD assessment[3]					X	X				
Chronic GVHD Assessment							X	X	X	X
Toxicity Evaluations		X	X	X	X	X				
Patient Diaries						To begin as outpatient				
Correlative Studies		X			X		X			
Assessment of Survival										X

1) Per institution practice guidelines: Includes Electrocardiogram, MUGA or Echocardiography, Pulmonary Function Testing.
2) Per institution practice guidelines: includes CBC with differential, serum chemistries, and infectious disease markers in pre-HCT period. CBC with differential, serum creatinine, AST, ALT, and total bilirubin measured thereafter.
3) Per institution practice guidelines: Assessment for acute GVHD will occur weekly through day 100. Following the "on study" period (see section 6.0) at minimum monthly assessments for GVHD (acute and chronic) are recommended. Assessments after day 100 are for exploratory secondary endpoints and thus not required observations.

7.0 ADVERSE EVENTS

7.1 Experimental Therapy

7.1.1 Contraindications

No contraindications to administration of this dietary supplement other than the exclusion criteria noted. Specifically, should an enrolled subject develop one of the exclusion criteria as listed in section 3.2 (ie active Clostridium difficile infection or IBD) after enrollment and initiation of the study, they will be allowed to continue on the treatment protocol. Development of acute GVHD is not considered a contraindication to continue with study protocol as long as the subject continues on an oral diet.

7.1.2 Interaction with other medications

As a dietary supplement and component of normal dietary intake, there are no anticipated interactions with other medications.

7.1.3 Adverse Reactions

No adverse reactions have been noted with the previous administration of this dietary supplement to a group of healthy volunteers (HUM00103995).

7.2 Adverse Event and Reporting Definitions

In the event of an adverse event, the first concern will be for the safety of the subject. Investigators are required to report any serious adverse event, whether expected or unexpected , and which is felt by the investigator to be reasonably or possibly related to or caused by the dietary supplement.All events meeting these criteria will be reported for the time period beginning with any amount of exposure to the dietary supplement through the protocol-defined follow-up period. Serious criteria, definitions, and guidance for reporting follow.

7.2.1 Adverse Event

An adverse event (AE) is any untoward medical occurrence in a patient receiving study treatment and which does not necessarily have a causal relationship with this treatment. An AE can be any unfavorable and unintended sign (including an abnormal laboratory finding), symptom, or disease temporally associated with the use of an experimental intervention, whether or not related to the intervention.

7.2.2 Serious Adverse Event

An adverse event is considered "serious" if, in the view of either the investigator it results in any of the following outcomes:

- Death
  If death results from (progression of) the disease, the disease should be reported as event (SAE) itself.
- A life-threatening adverse event
  An adverse even is considered 'life-threatening' if, in the view of either the investigator [or sponsor], its occurrence places the patient or subject at immediate risk of death. It does not include an adverse event that, had it occurred in a more severe form, might have caused death.
- Inpatient hospitalization or prolongation of existing hospitalization for > 24 hours.
- A persistent or significant incapacity or substantial disruption of the ability to conduct normal life functions
- A congenital anomaly/birth defect
- Important medical event

  Any event that may not result in death, be life-threatening, or require hospitalization may be considered serious when, based upon appropriate medical judgment, they may jeopardize the patient and may require medical or surgical intervention to prevent one of the outcomes listed in this definition of "Serious Adverse Event". Examples of such medical events include allergic bronchospasm requiring intensive treatment in an emergency room or at home; convulsions that do not result in inpatient hospitalization or the development of drug dependency or drug abuse.

7.2.3 Expected Adverse Events

An adverse event (AE) is considered "expected" if:

- For approved and marketed drugs or devices, those adverse events are described in the approved Package Insert (Label).
- For investigational new drugs or devices, those adverse events are described in the FDA Investigator's Brochure.
- In clinical research studies, information on expected adverse events is also summarized in the protocol and in the consent document. See section 8.0 for the list of expected adverse events related to the drug under study.

7.2.4 Unexpected Adverse Event

An adverse event (AE) is typically considered "unexpected" if it is not described in the Package Insert, Investigator's Brochure, in published medical literature, in the protocol, or in the informed consent document. As this study pertains to a commercially available dietary supplement containing resistant starch, it does not contain a package insert or investigator's brochure. Anticipated side effects are limited and listed in the informed consent. Therefore, AE not listed will not be listed as "unexpected" as long as they are consistent with adverse events typically anticipated for a usual transplant course. However, these events will be recorded to determine if they are occurring at an unusually high frequency.

7.2.5 CTCAE Term

(AE description) and grade: The descriptions and grading scales found in the NCI Common Terminology Criteria for Adverse Events (CTCAE) version 4.0 will be utilized for AE reporting. All appropriate treatment areas should have access to a copy of the CTCAE version 4.0. A copy of the CTCAE version 4.0 can be down loaded from the CTEP web site. (http://ctep.cancer.gov)

7.2.6 Attribution of the AE

The investigator or co-investigator is responsible for assignment of attribution.
Definite – The AE *is clearly related* to the study treatment
Probable – The AE *is likely related* to the study treatment
Possible – The AE *may be related* to the study treatment
Unlikely – The AE is doubtfully related to the study treatment
Unrelated – The AE is clearly NOT related to the study treatment

7.3 Reporting of Serious Adverse Events Associated with Potato-Starch produced by Bob's Red Mill® (IND 132208)

Event reporting for Bone Marrow Transplant Protocols can be complicated and confusing to investigators, data managers, and regulatory oversight bodies because patients typically develop numerous complications such as infections, chemotherapy-related organ damage, medication side effects, etc as part of the typical course of a bone marrow transplant and not related to the study therapy. Furthermore, transplant-related complications often occur both simultaneously and in series, as one complication leads to a series of additional downstream events, making time-sensitive reporting of events difficult. Therefore, a well-conceived event reporting plan will separate complications that might be seen with any transplant, from study-related events that are relevant to subject safety.

In order to achieve this goal, the DSM plan for this study will focus on rapid and specific identification and reporting of the following as SAEs:

a. Events which are serious and likely, probably or definitely related to the investigational component of study therapy.

b. Events occurring at unusual frequency or severity in study subjects compared to non-study subjects undergoing similar transplants.

c. Events resulting in death regardless of attribution.

d. Events that are serious and unexpected (unexpected is defined as not included in the study consent or the transplant consent.)

Therefore, we will not report as SAEs events that are expected and coincident with a typical transplant course unless they are either fatal or related to the investigational therapy.

7.4 Serious Adverse Event Reporting Guidelines

7.4.1 The Principal Investigator must be promptly notified as soon as reasonably possible by the study team of any event meeting the criteria and definition of a serious adverse event related to the dietary supplement, occurring during the study or within 7 days of the last administration of the study related treatment.

7.4.2 All serious adverse events that are definitely or probably related to potato starch produced by Bob's Red Mill® (IND 132208), and will be reported to the IRB per current institutional standards.

7.5 Adverse Event Form Reporting Guidelines

In addition to completing appropriate patient demographic and suspect medication information, the report should include the following information within the Event

- Description of the Adverse Event Form.
- Protocol description (and number, if assigned)
- Description of event, severity, treatment, and outcome if known
- Supportive laboratory results and diagnostics
- Investigator's assessment of the relationship of the adverse event to each investigational product and suspect medication

7.6 Reporting of Unanticipated Problems

There are types of incidents, experiences and outcomes that occur during the conduct of human subjects' research that represent unanticipated problems but are not considered adverse events. For example, some unanticipated problems involve social or economic harm instead of the physical

or psychological harm associated with adverse events. In other cases, unanticipated problems place subjects or others at increased risk of harm, but no harm occurs.

Upon becoming aware of any incident, experience, or outcome (not related to an adverse event) that may represent an unanticipated problem, the investigator should assess whether the incident, experience, or outcome represents an unanticipated problem. The incident, experience or outcomes is considered unanticipated if it meets all of the following criteria:

1. Unexpected (in terms of nature, severity, or frequency);
2. Related or possibly related to participation in the research; and
3. Suggests that the research places subjects or others at a greater risk of harm than was previously known or recognized.

If the investigator determines that the incident, experience, or outcome represents an unanticipated problem, the investigator must report it to the study team.

8.0 DRUG INFORMATION

This study investigates a food source and not a drug. While there are many potential sources for a potato-based starch, we have chosen to use potato starch produced by Bob's Red Mill® (IND 132208) because it is easily commercially available, economical and previously well tolerated in our healthy cohort.

Starches are regulated by the Food and Drug Administration. The FDA's Select Committee on GRAS (Generally Recognized as Safe) Substances published an opinion in 1979 that stated: "There is no evidence in the available information on unmodified or pregelatinized corn, high amylose corn, waxy maize, wheat, milo (also called grain sorghum starch), rice, potato, tapioca or arrowroot starch that demonstrates or suggests reasonable grounds to suspect a hazard to the public when they are used at levels that are now current or that might reasonably be expected in the future" (SCOGS 1979).

- Nutritional label:

DESCRIPTION | VIDEOS | RECIPES | RELATED PRODUCTS | REVIEWS | NUTRITIONAL INFO

Nutrient Facts
Serving Size: 1 Tbs(12g)
Servings Per Container: 56

	Amount Per Serving	% Daily Value
**Calories**	40	
**Calories from Fat**	0	
**Total Fat**	0 g	0 %
Saturated Fat	0 g	0 %
Trans Fat	0 g	0 %
**Cholesterol**	0 mg	0 %
**Sodium**	0 mg	0 %
**Total Carbohydrate**	10 g	3 %
Dietary Fiber	0 g	0 %
Sugars	0 g	N/A
**Protein**	0 g	0 %
**Vitamin A**		0 %
**Vitamin C**		0 %
**Calcium**		0 %
**Iron**		0 %

* Percent Daily Values (DV) are based on a 2000 calorie diet. Your daily values may be higher or lower depending on your calorie needs.

Ingredients:
potato starch

*Manufactured in a facility that also uses tree nuts and soy

- Classification – type of agent: Resistant starch
- Side effects: GI upset, bloating, flatulence
- Drug Interactions: None known
- Storage and stability: Avoid extremes of temperature. No need for refrigeration.
  Preparation and Dispensing:

  Mix pre-measured packet of potato starch (20 g) with water, juice, apple sauce or any other cool substance to make palatable. Starch substance should not be heated or warmed.
- Bone marrow transplant dieticians will work closely with subjects to individualize preparation of the starch so as to maximize tolerability of supplement for each subject.
- Administration:
  - No regulation for administration of starch with or without other food or medications.
  - **Doses should be administered twice daily but no limit as to how closely together doses can be given. For example, doses do NOT need to be given 12 hours apart and instead can be given at times that feel comfortable to the subject.**
  - Missed doses should be documented by care team while patient is inpatient.
  - Missed doses should be documented by the study coordinator in the electronic medical record during outpatient follow up .
  - Missed doses should simply be skipped with no need to make up this dose at a later time.
- Availability: Commercially Available

- Return and Retention of Study Drug:

As this is a commercially available food product, subjects can keep any remaining product at the end of study.

- Placebo: Will consist of a readily accessible (digestible) corn starch known as Amioca powder (Amylopectin) (Ingredion Inc.) The placebo has been safely used as a control in human clinical trials (NCT01939600, NCT01708694). Amioca powder will be identical in appearance and packaging as the resistant potato starch. Both food ingredients, placebo and resistant potato starch, are isocaloric (approximately 80 calories per day from either food ingredient).

9.0 CORRELATIVES/SPECIAL STUDIES

The goal of the planned laboratory correlative studies is to investigate the early impact of dietary supplementation with resistant starch on changes in the stool microbiome / metabolome, cellular (blood) immune subsets and plasma inflammatory markers in patients receiving allogeneic SCT. Changes in these parameters will be measured with patient's serving as their own internal controls by longitudinal assessment over time as outlined in section 6.2. Furthermore, we will compare to samples obtained from subjects not receiving the dietary intervention at identical time points as external controls. Obtaining samples serves as an important exploratory secondary endpoint and are thus optional but not considered a protocol deviation.

9.1 Sample Collection Guidelines

Samples will be procured by patients with the stool collection device and protocol as listed in Appendix C.

Stool samples will be collected at the following time points:

- Day -7 (+/- 3 day)
- Day -1 (+/- 3 day)
- Day +7 (+/- 3 days)
- At time of engraftment
- Q7-10 days from time of engraftment through day +100 (+/-7days)
- Day +100 (+/- 7 days)
- Day +130 (+/- 7 days)
- Day + 160 (+/- 7 days)

Correlative Studies – Blood Samples – will be collected at the following time points:

- Day -7 (+/- 3 day)
- At time of engraftment
- Day +100 (+/- 7 day)

9.2 Assay Methodology

Preserved specimens will analyzed for microbial nucleic acids to characterize the components of the stool microbiome. Additionally, key metabolic byproducts, including butyrate, will be identified in stool specimens using liquid chromatography and mass spectrometry. In subjects undergoing endoscopic biopsies for assessment for clinical GVHD (as part of routine SCT practice), we will obtain tissue blocks and perform immunohistochemistry for receptors to key metabolites. Other blood samples for patients co-enrolled on HUM00043287 may be analyzed to describe cellular immune subsets and plasma inflammatory markers (i.e. IL-6, TRN-α).

9.2 Specimen Banking

Patient samples collected for this study will be retained and processed at the University of Michigan Immunology Core Laboratory until analysis by other core laboratories and other collaborators. Specimens will be stored indefinitely or until they are used up. If future use is denied or withdrawn by the patient, the specimens will be destroyed but data obtained prior to revocation may still be utilized as part of pooled analysis.

Specimens being stored long-term for potential use not outlined in the protocol are subject to University Policy Governing Tissue Sample Collection, Ownership, Usage, and Disposition within all UMMS Research Repositories.

10.0 STATISTICAL CONSIDERATIONS

10.1 Study Design

This is a two-phase study designed to assess both the feasibility of administering a dietary supplement to patients receiving an allogeneic stem cell transplant, as well as the efficacy of a potato-based resistant starch for the prevention of acute GVHD.

Feasibility run in: We will initiate the study with 10 evaluable subjects as our initial feasibility cohort. We will conclude that administration of a resistant potato-based starch is feasible if 6 or more subjects are able to take 70% or more of their scheduled doses. We will also assess the effect of the dietary supplement on the structure of recipients' intestinal microbiome and its metabolites, particularly the short chain fatty acid (SCFA), butyrate. This portion of the study will be restricted to subject ≥ 18 years of age.

Phase II: Assuming our feasibility endpoint is reached, we will enroll a second cohort of 50 evaluable subjects,all of whom will receive a potato-based resistant starch as well as 10 additional evaluable subjects who will receive an iso-caloric, accessible starch and serve as biological contemporary controls. The studystatistician (Thomas Braun, Ph.D) will generate randomization lists in a 5:1 ratio (starch versus placebo) prior to the study in order to determine assignment of subjects to either the

potato-based

resistant starch OR to receive placebo (accessible corn starch). All subjects will be followed for the development of acute GVHD by day 100.

10.2 Sample Size and Accrual

The sample size of the feasibility cohort (10 patients) was selected primarily based upon the available patient resources at Rogel Cancer Center, as well as our desire to allocate as many patients as possible to the efficacy stage of the trial. Assuming a successful run in assessment of feasibility, an additional 50 patients will be assigned the dietary treatment to provide an initial statistical assessment of efficacy. Our overall sample size is based upon a 40% historical incidence of acute grade II-IV GVHD in matched related SCT by day 100 post SCT. With a total of 60 subjects (10 from feasibility run in and 50 from phase II) taking the protocol defined dietary supplement we will have 80% confidence with a Type I error rate of 5% to detect a reduction in acute GVHD from 40%to 25%.

The additional 10 randomized control patients will be used solely for hypothesis generating related to comparisons to the 50 patients in the experiment cohort with regard to the butyrate levels, intestinal microbiome composition, and Clostridium difficile infection, all of which are exploratory secondary aims, and are exploratory in nature.

Of note, this sample size of 70 total patients (10 feasibility cohort + 50 patients assigned to dietary treatment + 10 randomized control patients) refers to evaluable patients who are not replaceable. Since some subjects are replaceable and not evaluable per protocol, the actual number of patients who will be enrolled to the study will exceed the 70 evaluable patients.

10.3 Data Analyses Plans

Efficacy will be assessed through the estimated cumulative incidence of acute GVHD and a corresponding 95% confidence interval. The cumulative incidence will be computed such that death and relapse are treated as competing risks.

Analyses of data related to exploratory secondary aims will be purely descriptive, i.e. means and standard errors, and be used primarily for the design of future studies of potato-based starch.

10.4 Feasibility

Based on our experience with eligibility at the University of Michigan BMT program thus far, we anticipate approximately 20 patients per year to be eligible for the protocol. Assuming 50 % of such patients accrue to this protocol, we expect to complete the study within nine years of initiation.

11.0 DATA AND SAFETY MONITORING

This study will be monitored in accordance with the NCI approved University of Michigan Rogel Cancer Center Data and Safety Monitoring Plan.

The study team will meet quarterly or more frequently depending on the activity of the protocol. The discussion will include matters related to the safety of study participants (SAE/UaP reporting), validity and integrity of the data, enrollment rate relative to expectations, characteristics of participants, retention of participants, adherence to the protocol (potential or real protocol deviations) and data completeness. At these regular meetings, the protocol specific Data and Safety Monitoring Report form will be completed and signed by the Principal Investigator or by one of the co-investigators.

Data and Safety Monitoring Reports will be submitted to the University of Michigan Rogel Cancer Center Data and Safety Monitoring Committee (DSMC) on a quarterly basis for independent review.

12.0 REFERENCES

Nutritional information. Vol. 2016.

Atarashi, K., Tanoue, T., Oshima, K., Suda, W., Nagano, Y., Nishikawa, H., Fukuda, S., Saito, T., Narushima, S., Hase, K., Kim, S., Fritz, J.V., Wilmes, P., Ueha, S., Matsushima, K., Ohno, H., Olle, B., Sakaguchi, S., Taniguchi, T., Morita, H., Hattori, M. & Honda, K. (2013) Treg induction by a rationally selected mixture of Clostridia strains from the human microbiota. *Nature,* **500,** 232-236.

Brennan, T.V., Lin, L., Huang, X., Cardona, D.M., Li, Z., Dredge, K., Chao, N.J. & Yang, Y. (2012) Heparan sulfate, an endogenous TLR4 agonist, promotes acute GVHD after allogeneic stem cell transplantation. *Blood,* **120,** 2899-2908.

Chang, P.V., Hao, L., Offermanns, S. & Medzhitov, R. (2014) The microbial metabolite butyrate regulates intestinal macrophage function via histone deacetylase inhibition. *Proc Natl Acad Sci U S A,* **111,** 2247-2252.

Chen, Y., Zhao, Y., Cheng, Q., Wu, D. & Liu, H. (2015) The Role of Intestinal Microbiota in Acute Graft-versus-Host Disease. *J Immunol Res,* **2015,** 145859.

Choi, S.W., Braun, T., Chang, L., Ferrara, J.L., Pawarode, A., Magenau, J.M., Hou, G., Beumer, J.H., Levine, J.E., Goldstein, S., Couriel, D.R., Stockerl-Goldstein, K., Krijanovski, O.I., Kitko, C., Yanik, G.A., Lehmann, M.H., Tawara, I., Sun, Y., Paczesny, S., Mapara, M.Y., Dinarello, C.A., DiPersio, J.F. & Reddy, P. (2014) Vorinostat plus tacrolimus and mycophenolate to prevent graft-versus-host disease after related-donor reduced-intensity conditioning allogeneic haemopoietic stem-cell transplantation: a phase 1/2 trial. *Lancet Oncol,* **15,** 87-95.

Cummings, J.H., Beatty, E.R., Kingman, S.M., Bingham, S.A. & Englyst, H.N. (1996) Digestion and physiological properties of resistant starch in the human large bowel. *Br J Nutr,* **75,** 733-747.

Deeg, H.J. (2007) How I treat refractory acute GVHD. *Blood,* **109,** 4119-4126.

Ek, K.L., Wang, S., Copeland, L. & Brand-Miller, J.C. (2014) Discovery of a low-glycaemic index potato and relationship with starch digestion in vitro. *Br J Nutr,* **111,** 699-705.

Ferrara, J.L., Levine, J.E., Reddy, P. & Holler, E. (2009) Graft-versus-host disease. *Lancet,* **373,** 1550-1561.

Goldsmith, J.R. & Sartor, R.B. (2014) The role of diet on intestinal microbiota metabolism: downstream impacts on host immune function and health, and therapeutic implications. *J Gastroenterol,* **49,** 785-798.

Gomez-Almaguer, D., Ruiz-Arguelles, G.J., del Carmen Tarin-Arzaga, L., Gonzalez-Llano, O., Gutierrez-Aguirre, H., Cantu-Rodriguez, O., Jaime-Perez, J., Carrasco-Yalan, A. & Giralt, S. (2008) Alemtuzumab for the

treatment of steroid-refractory acute graft-versus-host disease. *Biol Blood Marrow Transplant,* **14,** 10-15.

Kaur, A., Rose, D.J., Rumpagaporn, P., Patterson, J.A. & Hamaker, B.R. (2011) In vitro batch fecal fermentation comparison of gas and short-chain fatty acid production using "slowly fermentable" dietary fibers. *J Food Sci,* **76,** H137-142.

Lee, S.J., Klein, J., Haagenson, M., Baxter-Lowe, L.A., Confer, D.L., Eapen, M., Fernandez-Vina, M., Flomenberg, N., Horowitz, M., Hurley, C.K., Noreen, H., Oudshoorn, M., Petersdorf, E., Setterholm, M., Spellman, S., Weisdorf, D., Williams, T.M. & Anasetti, C. (2007) High-resolution donor-recipient HLA matching contributes to the success of unrelated donor marrow transplantation. *Blood,* **110,** 4576-4583.

Levine, J.E., Logan, B., Wu, J., Alousi, A.M., Ho, V., Bolanos-Meade, J. & Weisdorf, D. (2010) Graft-versus-host disease treatment: predictors of survival. *Biol Blood Marrow Transplant,* **16,** 1693-1699.

MacMillan, M.L., DeFor, T.E. & Weisdorf, D.J. (2010) The best endpoint for acute GVHD treatment trials. *Blood,* **115,** 5412-5417.

Mathewson, N.D., Jenq, R., Mathew, A.V., Koenigsknecht, M., Hanash, A., Toubai, T., Oravecz-Wilson, K., Wu, S.-R., Sun, Y., Rossi, C., Fujiwara, H., Byun, J., Shono, Y., Lindemans, C., Calafiore, M., Schmidt, T.C., Honda, K., Young, V.B., Pennathur, S., Brink, M.v.d. & Reddy, P. (2016) Gut microbiome derived metabolites modulate intestinal epithelial cell damage and mitigate Graft-versus-Host Disease. *Nature Immunology*.

Nash, R.A., Pineiro, L.A., Storb, R., Deeg, H.J., Fitzsimmons, W.E., Furlong, T., Hansen, J.A., Gooley, T., Maher, R.M., Martin, P., McSweeney, P.A., Sullivan, K.M., Anasetti, C. & Fay, J.W. (1996) FK506 in combination with methotrexate for the prevention of graft-versus-host disease after marrow transplantation from matched unrelated donors. *Blood,* **88,** 3634-3641.

Pasquini MC, Z.X. (2014) Current uses and outcomes of hematopoietic stem cell transplantation: 2014 CIBMTR Summary Slides.

Peterson, L.W. & Artis, D. (2014) Intestinal epithelial cells: regulators of barrier function and immune homeostasis. *Nat Rev Immunol,* **14,** 141-153.

Raben, A., Tagliabue, A., Christensen, N.J., Madsen, J., Holst, J.J. & Astrup, A. (1994) Resistant starch: the effect on postprandial glycemia, hormonal response, and satiety. *Am J Clin Nutr,* **60,** 544-551.

Ratanatharathorn, V., Nash, R.A., Przepiorka, D., Devine, S.M., Klein, J.L., Weisdorf, D., Fay, J.W., Nademanee, A., Antin, J.H., Christiansen, N.P., van der Jagt, R., Herzig, R.H., Litzow, M.R., Wolff, S.N., Longo, W.L., Petersen, F.B., Karanes, C., Avalos, B., Storb, R., Buell, D.N., Maher, R.M., Fitzsimmons, W.E. & Wingard, J.R. (1998) Phase III study comparing methotrexate and tacrolimus (prograf, FK506) with methotrexate and cyclosporine for graft-versus-host disease prophylaxis after HLA-identical sibling bone marrow transplantation. *Blood,* **92,** 2303-2314.

SCOGS (1979) SCOGS Opinion: Starches, cereal starches, pregelatinized starch. US FDA, GRAS Substances Database.

Slavin, J.L. (2013) Carbohydrates, dietary fiber, and resistant starch in white vegetables: links to health outcomes. *Adv Nutr,* **4,** 351S-355S.

13.0 APPENDICES

APPENDIX A: KARNOFSKY PERFORMANCE SCALE

%	Description
100	Normal, no complaints, no evidence of disease
90	Able to carry on normal activity, minor symptoms of disease
80	Normal activity with effort, some signs of symptoms of disease
70	Cares for self (consistent with age), unable to carry on normal activity or do active work/school/play
60	Requires occasional assistance (beyond age-appropriate care), but is able to care for most of their needs
50	Requires considerable assistance and frequent medical care
40	Disabled, requires special care and assistance
30	Severely disabled, hospitalization is indicated although death is not imminent
20	Hospitalization is necessary, very sick, active support treatment is necessary
10	Moribund, fatal processes progressing rapidly

APPENDIX B: ACUTE GVHD ASSESSMENT GUIDELINES:

GVHD STAGING

Stage	Skin	Liver (bilirubin)	Gut (stool output/day)
0	No GVHD rash	< 2 mg/dl	Adult: < 500 ml/day<br>Child: < 10 ml/kg/day
1	Maculopapular rash<br>< 25% BSA	2-3 mg/dl	Adult: 500–999 ml/day<br>Child: 10 -19.9 ml/kg/day<br>Or persistent nausea, vomiting, or anorexia, with a positive upper GI biopsy.
2	Maculopapular rash<br>25 – 50% BSA	3.1-6 mg/dl	Adult: 1000-1500 ml/day<br>Child: 20 – 30 ml/kg/day
3	Maculopapular rash<br>> 50% BSA	6.1-15 mg/dl	Adult: >1500 ml/day<br>Child: > 30 ml/kg/day
4	Generalized erythroderma <u>plus</u> bullous formation and desquamation > 5% BSA	>15 mg/dl	Severe abdominal pain with or without ileus, or grossly bloody stool (regardless of stool volume).

- For GI staging: The "adult" stool output values should be used for patients ≥ 50 kg in weight.
- Use 3 day averages for GI staging based on stool output. If stool and urine are mixed, stool output is estimated to be 50% of total stool/urine mix.
- For stage 4 GI: the term "severe abdominal pain" will be defined as:
  (a) Pain control requiring institution of opioid use, or an increase in on-going opioid use, PLUS
  (b) Pain that significantly impacts performance status, as determined by the treating MD.
- If colon or rectal biopsy is +, but stool output is <500 ml/day (<10 ml/kg/day), then consider as GI stage 0.
- There is no modification of liver staging for other causes of hyperbilirubinemia (see appendix A).

Overall Clinical Grade:

Grade 0	No stage 1-4 of any organ
Grade I	Stage 1-2 rash and no liver or gut involvement
Grade II	Stage 3 rash, or Stage 1 liver involvement, or Stage 1 GI
Grade III	Stage 0-3 skin, with Stage 2-3 liver, or Stage 2-3 GI
Grade IV	Stage 4 skin, liver or GI involvement

APPENDIX C: STOOL COLLECTION PROCESS + INITIAL PROCESSING

Sampling Protocol – IN-PATIENT:
1. RN will remind you sample is due
2. Flush the toilet twice
3. Place wax paper on the water in the toilet bowl
4.

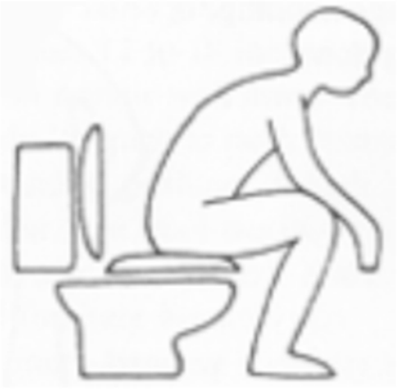

5. Collect a sample following instructions in kit and **SHAKE** to preserve. If stool is liquid, use syringe instead of spatula
6. Flush the toilet with the wax paper
7. Notify RN sample is ready
8. RN collects sample, labels with date and places in appropriate location for collection

Sampling Protocol – HOME:

1. Look at calendar, remember sample is due
2. Flush the toilet twice
3. Place wax paper on the water in the toilet bowl
4.

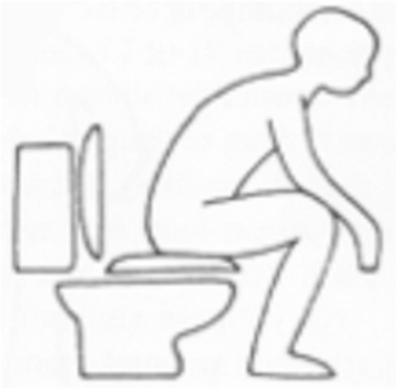

5. Collect a sample following instructions in kit and **SHAKE** to preserve. If stool is liquid, use syringe instead of spatula
6. Flush the toilet with the wax paper
7. Fill out the DATE on the label
8. Bring sample to NEXT clinic visit in a biohazard bag

From Sample Collection to MoBio Bead Plates

Sample Collection:

1. When collecting a sample, 'less is more.' Do not overload the yellow cap with fecal material. There will be plenty of DNA to analyze, even if the yellow cap is not densely packed.
2. Shake the tube very thoroughly. This is important for homogenizing the sample and to make sure that the DNA-preserving buffer is in contact with all of the sample.
3. Tube must be weighed prior to freezing. Scale is in research lab on 7W in Mott C&W Hospital. Weight should be recorded in log book to two decimal points. Sample should be weighed and placed in freezer within 24-48 hours after it is obtained.
4. For long term storage, freeze tubes vertically. This will make processing much easier and will ensure that all of the sample can be used.
    a. If performing SCFA analysis on the samples, freeze within 24 hours of collection.

Moving Samples to Bead Plates and SCFA Analysis Plates

- Use wide-bore (large orifice), aerosol barrier pipette tips for the initial transfer. Fisherbrand specialty tips (catalog #02-707-180) work well, and have the added bonus of being slightly longer than the genotek tubes, so that the pipettor does not need to reach into the tube, just the tip.
- Thaw tubes for a few minutes at room temperature. Store in the fridge if not processing immediately.
- Keep the 96-well bead plate on ice while transferring samples.
- Before taking the mat off the bead plate, label the corners A1 and H12 so that it is always put on in the same orientation, avoiding any contamination between wells.
- Make sure that you know the weight of each tube before removing and sample from it.

1. Check under the cap of the tube to make sure that there is not a large amount of fecal material stuck in the cap. If there is, consider discarding this sample.
2. With a pipette tip or a vortex on a very low setting, mix the sample.
    a. Do not incorporate fecal material that is stuck to the sides of the tube. This material has not been in the buffer, so integrity of the DNA in this sample is unknown.
3. Move 250µL-300µL into each well of the bead plate.
    a. When pipetting the sample, it can sometimes be helpful to tap the end of the tip against the bottom of the tube to help any chunks break up and get into the pipette tip.
4. Move ~1mL into each well of the SCFA analysis plate.
    a. If possible, the positions on the bead plate and the SCFA analysis plate should match. This does not have processing or interpretation implications, it just makes it easier to keep things organized.
5. Move the remaining sample to tubes for long-term storage.
    a. Try to get at least 250µL into each tube. If there is more, split that between the tubes.
    b. Sometimes tubes do not have enough sample to get any save vials.

c. We generally try to get 2 save vials. How many you want to create will depend on how you intend to use saved samples.

6. Freeze the plate until ready to extract DNA.

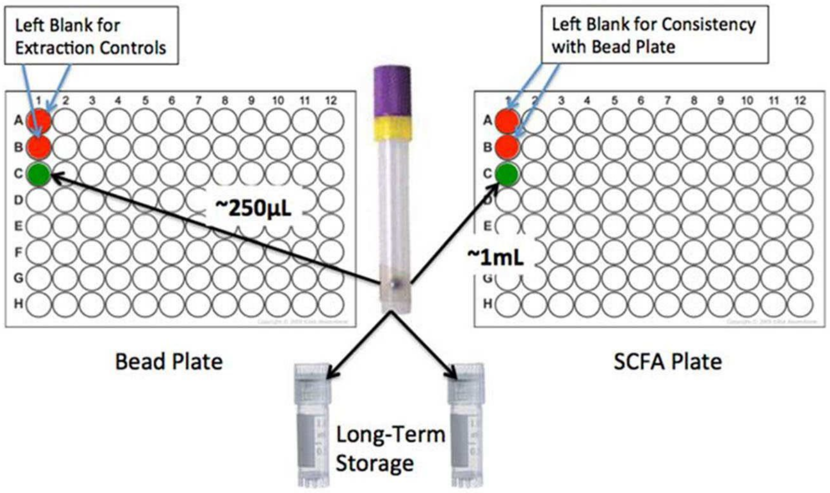

Supplementary Table 5 - The STORMS checklist. An editable version for adaptation and inclusion in publications is available from https://stormsmicrobiome.org

Number	Item	Recommendation	Item Source	Additional Guidance	Yes/No/NA	Comments or location in manuscript
**Abstract**						
1.0	Structured or Unstructured Abstract	Abstract should include information on background, methods, results, and conclusions in structured or unstructured format.	STORMS		Yes	abstract
1.1	Study Design	State study design in abstract.	STORMS	See 3.0 for additional information on study design.	Yes	abstract
1.2	Sequencing methods	State the strategy used for metagenomic classification.	STORMS	For example, targeted 16S by qPCR or sequencing, shotgun metagenomics, metatranscriptomics, etc.	Yes	This is currently in page 7 of results section and page 22 of methods section but we would be happy to add to the abstract as well.
1.3	Specimens	Describe body site(s) studied.	STORMS		Yes	Abstract (stool)
**Introduction**						
2.0	Background and Rationale	Summarize the underlying background, scientific evidence, or theory driving the current hypothesis as well as the study objectives.	STORMS		Yes	Introduction

2.1	Hypotheses	State the pre-specified hypothesis. If the study is exploratory, state any pre-specified study objectives.	STORMS		Yes	Introduction
**Methods**						
3.0	Study Design	Describe the study design.	STORMS	Observational (Case-Control, Cohort, Cross-sectional survey, etc.) or Experimental (Randomized controlled trial, Non-randomized controlled trial, etc.). For a brief description of common study designs see: DOI: 10.11613/BM.2014.022<br><br>If applicable, describe any blinding (e.g. single or double-blinding) used in the course of the study.	Yes	Methods
3.1	Participants	State what the population of interest is, and the method by which participants are sampled from that population. Include relevant information on physiological state of the subjects or stage in the life history of disease under study when participants were sampled.	STORMS	Examples of the population of interest could be: adults with no chronic health conditions, adults with type II diabetes, newborns, etc. This is the total population to whom the study is hoped to be generalizable to. The sampling method describes how potential participants were selected from that population.<br><br>If the participants are from a substudy of a larger study, provide a brief description of that study and cite that study.<br><br>Clearly state how cases and controls are defined.	Yes	Methods

				An example of relevant physiological state might be pre/post menopausal for a vaginal microbiome study; examples of stage in the life history of disease could be whether specimens were collected during active or dormant disease, or before or after treatment.		
3.2	Geographic location	State the geographic region(s) where participants were sampled from.	MIxS: geographic location (country and/or sea,region)	Geographic coordinates can be reported to prevent potential ambiguities if necessary.	Yes	methods
3.3	Relevant Dates	State the start and end dates for recruitment, follow-up, and data collection.	STORMS	Recruitment is the period in which participants are recruited for the study. In longitudinal studies, follow-up is the date range in which participants are asked to complete a specific assessment. Finally, data collection is the total period in which data is being collected from participants including during initial recruitment through all follow-ups.	Yes	methods

3.4	Eligibility criteria	List any criteria for inclusion and exclusion of recruited participants.	Modified STROBE	Among potential recruited participants, how were some chosen and others not? This could include criteria such as sex, diet, age, health status, or BMI.<br><br>If there is a primary and validation sample, describe inclusion/exclusion criteria for each.	Yes	methods
3.5	Antibiotics Usage	List what is known about antibiotics usage before or during sample collection.	STORMS	If participants were excluded due to current or recent antibiotics usage, state this here.<br><br>Other factors (e.g. proton pump inhibitors, probiotics, etc.) that may influence the microbiome should also be described as well.	Yes	Results section page 7 but happy to add to methods section as well
3.6	Analytic sample size	Explain how the final analytic sample size was calculated, including the number of cases and controls if relevant, and reasons for dropout at each stage of the study. This should include the number of individuals in whom microbiome sequencing was attempted and the number in whom microbiome sequencing was successful.	STORMS	Consider use of a flow diagram (see template at https://stormsmicrobiome.org/figures) . Also state sample size in abstract.<br><br>If power analysis was used to calculate sample size, describe those calculations.	Yes	methods
3.7	Longitudinal Studies	For longitudinal studies, state how many follow-ups were conducted, describe sample size at follow-up by group or condition, and discuss any loss to follow-up.	STORMS	If there is loss to follow-up, discuss the likelihood that drop-out is associated with exposures, treatments, or outcomes of interest.	Yes	methods

3.8	Matching	For matched studies, give matching criteria.	Modified STROBE	"Matched" refers to matching between comparable study participants as cases and controls or exposed / unexposed.<br><br>Indicate whether participants were individual or frequency matched and in what ratio were they matched (e.g. 1 case to 1 control).	N/A	
3.9	Ethics	State the name of the institutional review board that approved the study and protocols, protocol number and date of approval, and procedures for obtaining informed consent from participants.	STORMS		Yes	methods
4.0	Laboratory methods	State the laboratory/center where laboratory work was done.	STORMS	Provide a reference to complete lab protocols if previously published elsewhere such as on protocols.io. Note any modifications of lab protocols and the reason for protocol modifications.	N/A	
4.1	Specimen collection	State the body site(s) sampled from and how specimens were collected.	MIxS: sample collection device or method; host body site	Use terms from the Uber-anatomy Ontology (https://www.ebi.ac.uk/ols/ontologies/uberon) to describe body sites in a standardized format.	Yes	methods
4.2	Shipping	Describe how samples were stored and shipped to the laboratory.	STORMS	Include length of time from collection to receipt by the lab and if temperature control was used during shipping.	N/A	
4.3	Storage	Describe how the laboratory stored samples, including time between collection and storage and any preservation buffers or refrigeration used.	STORMS	State where each procedure or lot of samples was done if not all in the same place.	Yes	methods

				Include reagent/lot/catalogue #s for storage buffers.		
4.4	DNA extraction	Provide DNA extraction method, including kit and version if relevant.	MIxS: nucleic acid extraction	If any DNA quantification methods were used prior to DNA amplification or at the pooling step of library preparation, state so here.	Yes	methods
4.5	Human DNA sequence depletion or microbial DNA enrichment	Describe whether human DNA sequence depletion or enrichment of microbial or viral DNA was performed.	STORMS		Yes	methods
4.6	Primer selection	Provide primer selection and DNA amplification methods as well as variable region sequenced (if applicable).	MIxS: pcr primers		Yes	methods
4.7	Positive Controls	Describe any positive controls (mock communities) if used.	STORMS	If used, should be deposited under guidance provided in the 8.X items.	N/A	
4.8	Negative Controls	Describe any negative controls if used.	STORMS	If used, should be deposited under guidance provided in the 8.X items.	N/A	
4.9	Contaminant mitigation and identification	Provide any laboratory or computational methods used to control for or identify microbiome contamination from the environment, reagents, or laboratory.	STORMS	Includes filtering of reagents and other steps to minimize contamination. It is relevant to state whether the specimens of interest have low microbial load, which makes contamination especially relevant.	Yes	methods
4.10	Replication	Describe any biological or technical replicates included in the sequencing, including which steps were replicated between them.	STORMS	Replication may be biological (redundant biological specimens) or technical (aliquots taken at different stages of analysis) and used in	Yes	methods

				extraction, sequencing, preprocessing, and/or data analysis.		
4.11	Sequencing strategy	Major divisions of strategy, such as shotgun or amplicon sequencing.	MIxS: sequencing method	For amplicon sequencing (for example, 16S variable region), state the region selected. State the model of sequencer used.	Yes	methods
4.12	Sequencing methods	State whether experimental quantification was used (QMP/cell count based, spike-in based) or whether relative abundance methods were applied.	STORMS	These include read length, sequencing depth per sample (average and minimum), whether reads are paired, and other parameters.	Yes	methods
4.13	Batch effects	Detail any blocking or randomization used in study design to avoid confounding of batches with exposures or outcomes. Discuss any likely sources of batch effects, if known.	STORMS	Sources of batch effects include sample collection, storage, library preparation, and sequencing and are commonly unavoidable in all but the smallest of studies.	N/A	
4.14	Metatranscriptomics	Detail whether any mRNA enrichment was performed and whether/how retrotranscription was performed prior to sequencing. Provide size range of isolated transcripts. Describe whether the sequencing library was stranded or not. Provide details on sequencing methods and platforms.	STORMS	Provide details on any internal standards which may have been used as well as parameters and versions of any software or databases used.	N/A	
4.15	Metaproteomics	Detail which protease was used for digestion. Provide details on proteomic methods and platforms (e.g. LC-MS/MS, instrument type, column type, mass range, resolution, scan speed, maximum injection time, isolation window, normalised collision energy, and resolution).	STORMS	Provide details on any internal standards which may have been used as well as parameters and versions of any software or databases used.	N/A	

4.16	Metabolomics	Specify the analytic method used (such as nuclear magnetic resonance spectroscopy or mass spectrometry). For mass spectrometry, detail which fractions were obtained (polar and/or non-polar) and how these were analyzed. Provide details on metabolomics methods and platforms (e.g. derivatization, instrument type, injection type, column type and instrument settings).	STORMS	Provide details on any internal standards which may have been used as well as parameters and versions of any software or databases used.	Yes	Methods
5.0	Data sources/ measurement	For each non-microbiome variable, including the health condition, intervention, or other variable of interest, state how it was defined, how it was measured or collected, and any transformations applied to the variable prior to analysis.	MIxS: host disease status	State any sources of potential bias in measurements, for example multiple interviewers or measurement instruments, and whether these potential biases were assessed or accounted for in study design.<br><br>Use terms from a standardized ontology such as the Experimental Factor Ontology (https://www.ebi.ac.uk/efo/) to describe variables of interest in a standardized format.	Yes	Methods
6.0	Research design for causal inference	Discuss any potential for confounding by variables that may influence both the outcome and exposure of interest. State any variables controlled for and the rationale for controlling for them.	STORMS	For causal inference, this item refers to describing the assumptions that would be required to draw causal inferences from observational data. See Vujkovic-Cvijin, I., Sklar, J., Jiang, L. et al. Host variables confound gut microbiota studies of human disease. Nature 587, 448–454 (2020). https://doi.org/10.1038/s41586-020-2881-9 for more details on confounding in observational microbiome studies.	N/A	

				For example, hypothesized confounders may be controlled for by multivariable adjustment. Consider using a directed acyclic graph (DAG) to describe your causal model and justify any variables controlled for. DAGs can be made using www.dagitty.net.		
6.1	Selection bias	Discuss potential for selection or survival bias.	STORMS	Selection bias can occur when some members of the target study population are more likely to be included in the study/final analytic sample than others. Some examples include survival bias (where part of the target study population is more likely to die before they can be studied), convenience sampling (where members of the target study population are not selected at random), and loss to follow-up (when probability of dropping out is related to one of the things being studied).	N/A	
7.0	Bioinformatic and Statistical Methods	Describe any transformations to quantitative variables used in analyses (e.g. use of percentages instead of counts, normalization, rarefaction, categorization).	STORMS	If a variable is analyzed using different transformations, state rationale for the transformation and for each analyses which version of the variable is used.<br><br>In case of any complex or multistep transformations, give enumerated	Yes	methods

				instructions for reproducing those transformations.		
7.1	Quality Control	Describe any methods to identify or filter low quality reads or samples.	MIxS: sequence quality check	If samples were excluded based on quality or read depth, list the criteria used, the number of samples excluded, and the final sample size after quality control.	N/A	
7.2	Sequence analysis	Describe any taxonomic, functional profiling, or other sequence analysis performed.	MIxS: feature prediction; similarity search method		Yes	methods
7.3	Statistical methods	Describe all statistical methods.	Modified STROBE	Describe any statistical tests used, exploratory data analysis performed, dimension reduction methods/unsupervised analysis, alpha/beta metrics, and/or methods for adjusting for measurement bias.<br><br>If multiple statistical methods are possible, discuss why the methods used were selected.<br><br>If a multiple hypothesis testing correction method was used, describe the type of correction used.<br><br>State which taxonomic levels are analyzed.	Yes	methods

7.4	Longitudinal analysis	If the study is longitudinal, include a section that explicitly states what analysis methods were used (if any) to account for grouping of measurements by individual or patterns over time.	STORMS		Yes	methods
7.5	Subgroup analysis	Describe any methods used to examine subgroups and interactions.	STROBE		N/A	
7.6	Missing data	Explain how missing data were addressed.	STROBE	"Missing data" refers to participant measurements such as covariates, exposures, outcomes, or time points that should have been collected but were not, not to zeros in taxonomic abundance tables or data points not applicable to that observation.	N/A	
7.7	Sensitivity analyses	Describe any sensitivity analyses.	STROBE		N/A	
7.8	Findings	State criteria used to select findings for reporting.	STORMS	For example, false discovery rate with total number of tests, effect size threshold, significance threshold, microbes of interest.	N/A	
7.9	Software	Cite all software (including read mapping software) and databases (including any used for taxonomic reference or annotating amplicons, if applicable) used. Include version numbers.	Modified STREGA	Installed packages, add-ons or libraries should be stated and cited in addition to the software used.<br><br>All parameters employed that differ from the default of that software/version should be provided.<br><br>This is in addition to, not a replacement for, publishing of code as outlined in the section Reproducible Research.	Yes	methods

8.0	Reproducible research	Make a statement about whether and how others can reproduce the reported analysis.	STORMS	Any protected information that has been excluded or provided under controlled access should be listed along with any relevant data access procedures. "On request from authors" is not sufficiently detailed; formal data access procedures and conditions should be defined.<br><br>If data are unavailable, state so clearly.<br><br>Consider using a specialized rubric for reproducible research (such as: https://mbio.asm.org/content/9/3/e00525-18.short).<br><br>Consider preregistering the study protocol (such as on osf.io or https://plos.org/open-science/preregistration/).	Yes	Data availability page 26
8.1	Raw data access	State where raw data may be accessed including demultiplexing information.	STORMS	Robust, long-term databases such as those hosted by NCBI and EBI are preferred. If using a private repository, provide rationale.	Yes	Data availability page 26
8.2	Processed data access	State where processed data may be accessed.	STORMS	Unfiltered data should be provided.<br><br>Robust, long-term databases such as those hosted by NCBI and EBI-EMBL are preferred. Repositories like zenodo (https://zenodo.org/) or publisso (https://www.publisso.de/en/working-for-you/doi-service/)	yes	Data availability page 26

				can be used to provide a DOI and long-term storage for processed datasets, even those which cannot be published openly.		
8.3	Participant data access	State where individual participant data such as demographics and other covariates may be accessed, and how they can be matched to the microbiome data.	STORMS	If re-categorized, transformed, or otherwise derived variables were used in the analysis, these variables or code for deriving them should be provided.<br><br>Examples of how participant data can be matched to microbiome data are: using the same set of anonymized identifiers, or using different anonymized identifiers but providing a map.<br><br>Provided data should be sufficient to independently replicate the current analysis.	Yes	Data availability page 26
8.4	Source code access	State where code may be accessed.	STORMS	If a standard or formalized workflow was employed, reference it here.	Yes	Methods (MaLiAmPi)
8.5	Full results	Provide full results of all analyses, in computer-readable format, in supplementary materials.	STORMS	For example, any fold-changes, p-values, or FDR values calculated, provided as a spreadsheet.<br><br>Use a machine-readable, plain-text format such as csv or tsv.	Yes	Methods and supplement
**Results**						

9.0	Descriptive data	Give characteristics of study participants (e.g. dietary, demographic, clinical, social) and information on exposures and potential confounders.	STROBE	Typically reported in a table included in the paper or as a supplementary table. Indicate number of participants with missing data for each variable of interest.<br><br>This includes environmental and lifestyle factors that may affect the relationship between the microbiome and the condition of interest. Participant diet and medication use should be summarized, if known.<br><br>At minimum, age and sex of all participants should be summarized.	Yes	Results
10.0	Microbiome data	Report descriptive findings for microbiome analyses with all applicable outcomes and covariates.	STORMS	This includes measures of diversity as well as relative abundances. These descriptive findings should be reported both for the sample overall and for individual groups.	Yes	Results
10.1	Taxonomy	Identify taxonomy using standardized taxon classifications that are sufficient to uniquely identify taxa.	STORMS	If not using full taxonomic hierarchy, make sure it is clear whether names stated are species, genera, family, etc.<br><br>Italicize genus/species pairs. Consult journal guidelines or standardized references on taxonomic nomenclature. For instance, https://wwwnc.cdc.gov/eid/page/scientific-nomenclature	Yes	Methods

10.2	Differential abundance	Report results of differential abundance analysis by the variable of interest and (if applicable) by time, clearly indicating the direction of change and total number of taxa tested.	STORMS	If there are more than two groups, include omnibus (multigroup) test results if applicable to the research question.<br><br>If applicable, reported effect sizes should include a measure of uncertainty such as the confidence interval.	Yes	methods
10.3	Other data types	Report other data analyzed--e.g. metabolic function, functional potential, MAG assembly, and RNAseq.	STORMS		N/A	
10.4	Other statistical analysis	Report any statistical data analysis not covered above.	STORMS	This could include subgroup analysis, sensitivity analyses, and cluster analysis.<br><br>Visualizations should be easily interpretable and colorblind-friendly. The caption and/or main text should provide a detailed description of visualizations for visually-impaired readers.	Yes	Methods and supplement

Discussion

11.0	Key results	Summarise key results with reference to study objectives	STROBE		Yes	Discussion

12.0	Interpretation	Give a cautious overall interpretation of results considering objectives, limitations, multiplicity of analyses, results from similar studies, and other relevant evidence.	STROBE	Define or clarify any subjective terms such as "dominant," "dysbiosis," and similar words used in interpretation of results.<br><br>When interpreting the findings, consider how the interpretation of the findings may be summarized or quoted for the general public such as in press releases or news articles.<br><br>If causal language is used in the interpretation (such as "alters," "affects," "results in," "causes," or "impacts"), assumptions made for causal inference should be explicitly stated as part of 6.0 and 13.0.<br><br>Distinguish between function potential (ie inferred from metagenomics) and observed activity (ie metatranscriptomic, metabolomic, proteomic) if discussing microbial function.	Yes	Discussion
13.0	Limitations	Discuss limitations of the study, taking into account sources of potential bias or imprecision.	STROBE	Also consider limitations resulting from the methods (especially novel methods), the study design, and the sample size.	Yes	Discussion
13.1	Bias	Discuss any potential for bias to influence study findings.	STORMS	May include sampling method, representativeness of study participants, or potential confounding.	N/A	

13.2	Generalizability	Discuss the generalisability (external validity) of the study results	STROBE	To what populations or other settings do you expect the conclusions to generalize?	Yes	Discussion
14.0	Ongoing/future work	Describe potential future research or ongoing research based on the study's findings.	STORMS		Yes	Discussion
**Other information**						
15.0	Funding	Give the source of funding and the role of the funders for the present study and, if applicable, for the original study on which the present article is based	STROBE		yes	Acknowledgments page 26
15.1	Acknowledgements	Include acknowledgements of those who contributed to the research but did not meet critera for authorship.	STORMS	For general guidelines on authorship, see http://www.icmje.org and https://www.elsevier.com/authors/journal-authors/policies-and-ethics/credit-author-statement	N/A	
15.2	Conflicts of Interest	Include a conflicts of interest statement.	STORMS		Yes	Editorial checklist
16.0	Supplements	Indicate where supplements may be accessed and what materials they contain.	STORMS		Yes	
17.0	Supplementary data	Provide supplementary data files of results with for all taxa and all outcome variables analyzed. Indicate the taxonomic level of all taxa.	STORMS	Depending on the analysis performed, examples of the supplemental results included could be mean relative abundance, differential abundance, raw p-value, multiple hypothesis testing-adjusted p-values, and standard error.<br><br>All discussed taxa should include the taxonomic level (e.g. class, order, genus).	Yes	
