# Supplementary material for: Feasibility of a dietary intervention to modify gut microbial metabolism in patients with hematopoietic stem cell transplantation
Source: Nat Med. 2023 Oct 19;29(11):2805–13. doi: 10.1038/s41591-023-02587-y (PMC10667101; doi:10.1038/s41591-023-02587-y)
Supplement: Supplementary file 2 — Reporting Summary [file 41591_2023_2587_MOESM2_ESM.pdf]

nature portfolio

Corresponding author(s): Mary Riwes
Pavan Reddy

Last updated by author(s): August 28, 2023

Reporting Summary

Nature Portfolio wishes to improve the reproducibility of the work that we publish. This form provides structure for consistency and transparency in reporting. For further information on Nature Portfolio policies, see our Editorial Policies and the Editorial Policy Checklist.

Statistics

For all statistical analyses, confirm that the following items are present in the figure legend, table legend, main text, or Methods section.

n/a	Confirmed	
☐	☒	The exact sample size (*n*) for each experimental group/condition, given as a discrete number and unit of measurement
☐	☒	A statement on whether measurements were taken from distinct samples or whether the same sample was measured repeatedly
☐	☒	The statistical test(s) used AND whether they are one- or two-sided<br>*Only common tests should be described solely by name; describe more complex techniques in the Methods section.*
☐	☒	A description of all covariates tested
☐	☒	A description of any assumptions or corrections, such as tests of normality and adjustment for multiple comparisons
☐	☒	A full description of the statistical parameters including central tendency (e.g. means) or other basic estimates (e.g. regression coefficient) AND variation (e.g. standard deviation) or associated estimates of uncertainty (e.g. confidence intervals)
☐	☒	For null hypothesis testing, the test statistic (e.g. *F*, *t*, *r*) with confidence intervals, effect sizes, degrees of freedom and *P* value noted<br>*Give P values as exact values whenever suitable.*
☒	☐	For Bayesian analysis, information on the choice of priors and Markov chain Monte Carlo settings
☐	☒	For hierarchical and complex designs, identification of the appropriate level for tests and full reporting of outcomes
☒	☐	Estimates of effect sizes (e.g. Cohen's *d*, Pearson's *r*), indicating how they were calculated

Our web collection on statistics for biologists contains articles on many of the points above.

Software and code

Policy information about availability of computer code

Data collection	no software was used
Data analysis	Python 3.9 with umaplearn, statmodels and scikit-learn libraries were used for data analysis, with all parameters noted in the methods and text, defaults otherwise. Visualizations were accomplished with the matplotlib and seaborn libraries.

For manuscripts utilizing custom algorithms or software that are central to the research but not yet described in published literature, software must be made available to editors and reviewers. We strongly encourage code deposition in a community repository (e.g. GitHub). See the Nature Portfolio guidelines for submitting code & software for further information.

Data

Policy information about availability of data

All manuscripts must include a data availability statement. This statement should provide the following information, where applicable:

- Accession codes, unique identifiers, or web links for publicly available datasets
- A description of any restrictions on data availability
- For clinical datasets or third party data, please ensure that the statement adheres to our policy

Data in this manuscript have been presented where possible in aggregated form. All metabolite data generated in the preparation of this manuscript are available at https://doi.org/10.5281/zenodo.8015340. All microbiome data (16S rRNA amplicons) generated in the preparation of this manuscript have been deposited in NCBI's

Sequence Read Archive, with accession number (BioProejct ID) PRJNA953087. This study was prospectively registered. The clinical trial identifier is NCT02763033. It is also under FDA IND 132208. The pilot feasibility 10 patient study presented in this manuscript is the initial part of a phase II randomized trial currently enrolling 60 additional patients to determine clinical impact on acute GVHD.

Research involving human participants, their data, or biological material

Policy information about studies with human participants or human data. See also policy information about sex, gender (identity/presentation), and sexual orientation and race, ethnicity and racism.

Reporting on sex and gender	Gender: 6 males, 4 females<br>Sex: 6 males, 4 females<br>sex- and gender- based analyses were not performed given our small sample size of ten patients
Reporting on race, ethnicity, or other socially relevant groupings	N/A
Population characteristics	Age: The median age was 57 years (range 52-62 years) Diagnoses: 3 myelodysplastic syndrome (MDS); 2 acute myeloid leukemia (AML); 4 B-acute lymphoblastic lymphoma (B-ALL); 1 follicular lymphoma Treatment: All ten subjects received Resistant Potato Starch (RPS) per protocol. All ten subjects received HLA-matched related donor T-cell replete allogeneic hematopoietic stem cell transplantation with myeloablative conditioning, with standard graft versus host disease prophylaxis with tacrolimus and methotrexate as well as standard antibiotic prophylaxis with levaquin, and standard neutropenic fever treatment with IV cefepime (90%) or IV vancomycin along with IV aztreonam (10%). Acyclovir was used for viral prophylaxis and fluconazole for fungal prophylaxis in all participants.
Recruitment	All adults undergoing HLA-matched T cell replete related donor allogeneic hematopoeitic stem cell transplantation with myeloablative conditioning at the University of Michigan were recruited during their pre-transplant clinic visits. Participants were fully informed of the study and signed the consent form before any study procedures.
Ethics oversight	The trial was reviewed by the University of Michigan Protocol Review Committee, and has been approved by the Institutional Review Board.

Note that full information on the approval of the study protocol must also be provided in the manuscript.

Field-specific reporting

Please select the one below that is the best fit for your research. If you are not sure, read the appropriate sections before making your selection.

☒ Life sciences ☐ Behavioural & social sciences ☐ Ecological, evolutionary & environmental sciences

For a reference copy of the document with all sections, see nature.com/documents/nr-reporting-summary-flat.pdf

Life sciences study design

All studies must disclose on these points even when the disclosure is negative.

Sample size	This translational study has a sample size of 10 patients to test impact of resistant potato starch (RPS) as a prebiotic on the microbiome and microbial metabolites and assess clinical feasibility in allogeneic hematopoetic stem cell trasplantaion. The study presented in this manuscript is the first part of a two-phase study. The first phase presented here assessed feasibility of administration of RPS to 10 subjects. Feasibility is defined as ability to take 70% or more of scheduled doses in 60% or more of patients. The second phase of the study which is still ongoing will assess efficacy of RPS in preventing acute graft versus host disease (GVHD) in 60 subjects. This sample size was chosen based on 80% confidence with a Type I error rate of 5% to detect a reduction in acute GVHD from 40% to 25%.
Data exclusions	no data was excluded
Replication	Microbial metabolite, microbiome, and clinical data analyses are able to be replicated as they were analyzed independently by different experts and our statistician three times providing the same results. All metabolite and microbiome (16S rRNA amplicons) data have been made public (DOI and BioProject ID provided above) and all analyses were accomplished with open-source typically used Python data science packages.
Randomization	no randomization as this is a single arm feasibility study
Blinding	no blinding as this is a single arm feasibility study

Reporting for specific materials, systems and methods

We require information from authors about some types of materials, experimental systems and methods used in many studies. Here, indicate whether each material, system or method listed is relevant to your study. If you are not sure if a list item applies to your research, read the appropriate section before selecting a response.

Materials & experimental systems

n/a	Involved in the study
☒	☐ Antibodies
☒	☐ Eukaryotic cell lines
☒	☐ Palaeontology and archaeology
☒	☐ Animals and other organisms
☐	☒ Clinical data
☒	☐ Dual use research of concern
☒	☐ Plants

Methods

n/a	Involved in the study
☒	☐ ChIP-seq
☒	☐ Flow cytometry
☒	☐ MRI-based neuroimaging

Clinical data

Policy information about clinical studies

All manuscripts should comply with the ICMJE guidelines for publication of clinical research and a completed CONSORT checklist must be included with all submissions.

Clinical trial registration

NCT02763033. This study was prospectively registered before the start of patient enrollment: NCT02763033, FDA IND 132208. To clarify, the pilot feasibility 10 patient study presented in this manuscript is the initial part of a phase II randomized trial currently enrolling 60 additional patients to determine clinical impact on acute GVHD.

Study protocol

full protocol is attached

Data collection

From April 26, 2017 to September 30, 2018, 10 adults undergoing HLA matched, related-donor myeloablative allogeneic hematopoeitic stem cell transplantation (allo-HCT) were recruited. Stool and blood specimens were collected from the study participants at baseline prior to conditioning (day -7), nadir (~day 5-7), engraftment (~day 14), and day 100. Of note, if patients were able to provide weekly samples, then stool was collected and analyzed weekly. Samples were collected in the hospital or druing clinic visits. Following processing, stool and blood specimens were stored at -80C. Feasibility data was collected daily when patients were hospitalized for transplant and during outpatient visits after discharge through day 100 after transplant.

Outcomes

The primary objective was to test the feasibility of administering RPS to allo-HCT recipients and its effect on the structure of the patients' intestinal microbiome and its metabolites. We hypothesized that RPS would be feasible to administer and increase stool butyrate levels as a byproduct of microbial metabolism. An exploratory objective was to longitudinally evaluate plasma metabolites in recipients of RPS compared to historical controls. The primary endpoint was met. Feasibility met the preset goal of adherence to scheduled dosages and the butyrate levels were significantly higher while participants were on RPS as compared to when they were not on RPS ($p < 0.0001$). Furthermore, we observed longitudinal changes in plasma metabolites post allo-HCT compared to baseline independent of whether allo-HCT recipients received RPS ($p < 0.0001$). In RPS recipients, the dominant plasma metabolites were, however, much more stable across timepoints when compared to historic controls suggesting a greater equilibrium in their production and consumption.
